# A deep memory bare-bones particle swarm optimization algorithm for single-objective optimization problems

**DOI:** 10.1371/journal.pone.0284170

**Published:** 2023-06-02

**Authors:** Yule Sun, Jia Guo, Ke Yan, Yi Di, Chao Pan, Binghu Shi, Yuji Sato

**Affiliations:** 1 School of Information Engineering, Hubei University of Economics, Wuhan, China; 2 Hubei Internet Finance Information Engineering Technology Research Center, China; 3 China Construction Third Engineering Bureau Installation Engineering Co., Ltd., Wuhan, China; 4 Faculty of Computer and Information Sciences, Hosei University, Tokyo, Japan; Menoufia University, EGYPT

## Abstract

A deep memory bare-bones particle swarm optimization algorithm (DMBBPSO) for single-objective optimization problems is proposed in this paper. The DMBBPSO is able to perform high-precision local search while maintaining a large global search, thus providing a reliable solution to high-dimensional complex optimization problems. Normally, maintaining high accuracy while conducting global searches is an important challenge for single-objective optimizers. Traditional particle swarms optimizers can rapidly lose the diversity during iterations and are unable to perform global searches efficiently, and thus are more likely to be trapped by local optima. To address this problem, the DMBBPSO combines multiple memory storage mechanism (MMSM) and a layer-by-layer activation strategy (LAS). The MMSM catalyzes a set of deep memories to increase the diversity of the particle swarm. For every single particle, both of the personal best position and deep memories will be used in the evaluation process. The LAS enables the particle swarm to avoid premature convergence while enhancing local search capabilities. The collaboration between MMSM and LAS enhances the diversity of the particle swarm, which in turn enhances the robustness of the DMBBPSO. To investigate the optimization ability of the DMBBPSO for single-objective optimization problems, The CEC2017 benchmark functions are used in experiments. Five state-of-the-art evolutionary algorithms are used in the control group. Finally, experimental results demonstrate that the DMBBPSO can provide high precision results for single-objective optimization problems.

## Introduction

Single-objective optimization problem aims to select the optimal solution from all alternatives of a problem, which is still a very significant problem for research. It can be applied to practical problems in various fields, such as engineering optimization problems and scientific applications, which are usually computationally expensive and complex. The main methods for solving single-objective optimization problems are genetic algorithm, ant colony algorithm, particle swarm algorithm, and so on. Among them, the particle swarm optimizer (PSO) [1] is favored by researchers for its simple structure and fast running speed. The PSO is firstly proposed by Eberhart and Kennedy in 1995, which is an evolutionary computation algorithm. The PSO is originally motivated by the regularity of flocks birds and schools fish, which has the advantages of easy understanding, high accuracy and easy implementation. Since the PSO was proposed, it has received wide attention by plenty researchers. It has been used in dealing with nonlinear non-stationary problems and in real-world engineering problems. Such as power system anomaly detection [2], path planning [3], data clustering [4], image segmentation [5, 6], networks [7] and other fields.

In summary, a number of researches have shown that the optimal particle swarm algorithm has been well used in various aspects, however, the PSO algorithm and its many variants have problems in different aspects. For example, due to insufficient search extent, it is prone to be trapped into local optimum thus cannot guarantee to obtain the global optimum. In the meanwhile, premature maturity leaves the performance of the PSO algorithm to be optimized. Therefore, the PSO shows great prospect for further research.

As technology advances, researchers need to solve single-objective optimization problems in higher dimensions. Traditional optimization algorithms have the disadvantages of long solution time and low solution accuracy when facing high-dimensional optimization problems. To address these shortcomings, a deep memory bare-bones PSO (DMBBPSO) algorithm is introduced in this study. According to the intrinsic structure of the particles, the DMBBPSO has enhanced the update strategy by assigning multiple layers of memory to the particle population, which forms an innovative perspective.

The DMBBPSO is composed of two main modules: a multiple memory storage mechanism (MMSM) and a Layer-by-layer activation strategy (LAS). The MMSM enables an extra memory space for all particles. In each iteration, deep memories of particles are engaged in the evolution, thus the diversity of the particle is increased. The LAS ensures that the particle swarm is capable of using past positions to refine past choices. Main contributions of this work are reflected in following aspects:

1. The efficient deep memory topology enables the algorithm to choose its own evolutionary direction, and to refine past choices without human interaction.
2. Inspired by the social structure of wild championess, outdated information will be removed from memory spaces in a timely manner. This strategy ensures the accuracy and efficiency of the search.

The rest of this paper is orginized below: Section 2 presents the literature review; Section 3 presents the problem definitions and the proposed algorithm; Section 4 introduce the experimental settings and results analysis; at last, the conclusions of this work are shown in Section 5.

## Literature review

Jafari-Asl [8] proposes an optimization-simulation approach based on the PSO to get the optimum location and settings of pressure-reducing valves. Based on the PSO, Tan [9] proposes a PSO variant to improve the learning hyper-parameters of CNNS for skin lesion segmentation and difficult diverse image segmentation. Zhang [10] designed a PSO variant to extract effective spatial–temporal characteristics from the spectrogram inputs for sound classification tasks. Fernandes [11] proposes a novel quantum-behaved PSO used to get safe and efficient routes of mobile robotic vehicles in complex environment. Singh [12] proposes a hybrid algorithm with PSO to solve transportation problems.

Traditional PSO methods has some shortcomings as well, such as it will fall into local optimum easily, poor optimization processing for discrete problems and so on. To solve these problems, many scholars proposed the variants of PSO from various per-spectives such as Topology [13, 14] and updating strategies [15], improving learning strategies [16], and combination with other algorithms [17].

To increase the probability of finding the optimal solution, Li [18] proposes historical memory-based PSO (HMPSO), which preserve the particles’ information of the distribution of promising pbests in the history. Then, the best candidate position will be selected from the historical memory, the current pbests of the particle, and the gbest of the swarm.

In order to avoid falling into a local optimum, Tian [5] proposes Modified PSO (MPSO). The MPSO uses logistic graphs to generate particles that distributing uniformly and uses sigmoid-like inertia weights to enable PSO to employ inertia weight between linearly decreasing strategies and nonlinearly decreasing strategies adaptively. The MPSO has been proved to be efficient and effective in the tasks of standard image segmentation.

Karim [19] proposes modified PSO with effective guides (MPSOEG), which uses multiple learning strategies to create multiple exemplars instead of the self-cognitive and social components. This approach is used to improve the performance when dealing with complex optimization problems. In this study, the optimal guide creation (OGC) module is introduced, which can explore the particles at a lower computational cost. In the OGC, just the two nearest neighbors of global best particle will be considered and a diversity enhancement solution will be used to avoid premature convergence. Xu [20] proposes a parameter-free PSO integrating a reinforcement learning method. During its iteration, each particle will choose the optimal topology under the control of Q-learning (QL). The proposed strategy has been proved to be more superior compared with some methods.

Wang [21] proposes a cooperating PSO with depth first search strategy, which has better search capability in solving multimodal optimization problems. Li proposes [15] a new variant PSO, which uses novel competition and cooperation strategies to update the information of particles. The diversity of the swarm is enhanced, and it had a positive impact on the performance of PSO. Liu [22] designs an adaptive weighting strategy, which has the distinguishing feature of enhancing the convergence rate. Zhang [23] proposes a variant of BBPSO to solve the path planning problem for mobile robots.

In 2003, Kennedy [24] proposed the bare bones PSO (BBPSO), which eliminated the velocity term, and used random numbers which obedience to Gaussian distribution to update the positions of particles. It is a simple form of the particle swarm optimizer. The BBPSO has been proved to be outstanding to improve search efficiency and accuracy and has used successfully in constraint optimal issues, power system regulation and control, and data mining.

The BBPSO eliminated the velocity term, and used random numbers which obedience to Gaussian distribution to update the positions of the particles. The candidate position of particles will be update with Eq 1:

$$\begin{array}{c} x_{id}\left(t+1\right)=N(\frac{p_{id}\left(t\right)+p_{gd}\left(t\right)}{2},\left|p_{id}\left(t\right)-p_{gd}\left(t\right)\right|) \end{array}$$ (1)

where *x*_*id*_(*t* + 1) is the new position of the *id*th particle in the (*t* + 1)th iteration, *p*_*id*_(*t*) is the personal best position of the *id*th particle in the *t*th iteration, *p*_*gd*_(*t*) is the global best position of the *t*th iteration. In the *d* dimension search area, with a mean *μ* = (*p*_*id*(*t*) + *p*_*gd*(*t*))/2, and a standard deviation *σ* = |*p*_*id*(*t*) − *p*_*gd*(*t*)| of Gaussian distribution, the position of the *i*th particle will be updated. However, the BBPSO is not very effective in dealing with some multi-peaked problems and may be caught in a local optimum. In order to strengthen the capability of BBPSO, many researchers have made improvements on it.

Based on the BBPSO, a nominalized bare bones PSO algorithm [25] is proposed to solve the Traveling Salesman Problem, which has shown good performances. Zhang [26] designed a cooperative coevolutionary bare-bones PSO for the design of large-scale supply chain networks with uncertainty.

In order to avoid getting trapped in a local optimum and increase the performance of the BBPSO, Campos designed a new version of BBPSO named BBPSO with scale matrix adaptation (SMA-BBPSO), where a t-distribution is used to select a specific position of the particular. To improve the possibility of obtaining the best position of a particle, the strategy includes a rule for adjusting the scaling matrix. The experiments proved the effectiveness of proposed approach [27]. Guo proposes the pair-wise bare bones PSO (PBBPSO), which introduced a pair-wise strategy to the BBPSO. The strategy can improve the diversity of the swarm and avoid the excessively premature convergence. The particle pair will be chosen randomly at first. And they will be divided into two groups. The one that has a better position will be at leadership group and the other will be in the follower group. These two groups will be updated in different rules. In order to verify the better capability of PBBPSO compared to other optimization algorithm, Guo used a set of well-known benchmark functions in this study [28]. To alleviate the problem of premature convergence, Guo [29] designs a dynamic allocation bare bones PSO (DABBPSO). There are two groups which named as main group (MG) and ancillary group (AG) in this algorithm, which will work together to get the gbest. The focus of MG is to mine and try to find the optimal point in the current local optimum, the aim of AG is to explore the research area and give the whole swarm more chances to get rid of the local optimum. The optimization capability of the proposed algorithm is confirmed by the experimental results. Guo [30] proposes FHBBPSO, which introduce a fission strategy and a fusion strategy. They cooperate to obtain the theoretical optimal value. The fission strategy used to split the search space and the fusion strategy used to narrow the search space. The central group will gradually absorb the marginal groups and eventually merge into one group. According to the results of the test on the CEC2014, the proposed approach was confirmed to be an optimization algorithm with excellent competitiveness when solving single-objective problems.

## Materials and methods

The deep memory bare-bones PSO (DMBBPSO) algorithm is introduced in this section. The DMBBPSO is consisted of a multiple memory storage mechanism and a Lay-er-by-layer activation strategy. The efficient deep memory topology enables the algorithm to choose its own evolutionary direction, and to refine past choices without human inter-action.

### Multiple memory storage mechanism

The multiple memory storage mechanism (MMSM) is designed to enrich the diversity of the particle swarm. In MMSM, an extra storage is used to record the second best posi-tion of each particle. Second best positions engage in the evolution to catalyze the global search of the particle swarm, which realize the self-correction of the particle swarm. Spe-cifically, the candidate position of a particle is calculated by Eq 2:

$$\begin{array}{c} \begin{array}{c} \gamma =(m\_p\_p{\left(m,i\right)}^{t}+Gbest^{t})/2\\ \delta =|m\_pt\_p{\left(m,i\right)}^{t}+Gbest^{t}|\\ candidate\_x{\left(m,i\right)}^{t+1}=N\left(\gamma ,\delta \right) \end{array} \end{array}$$ (2)

where *m*_*p*_*p* is the memory list of the *i*th particle in the *t*th iteration, *candidate*_*x*(*m*, *i*)^*t*+1^ is the candidate position for the *i*th particle in the (*t* + 1)th iteration, *Gbestt* is the best position of the particle swarm in the *t*th iteration, *m* is the depth of the memory space, *N*(*γ*, *δ*) is the Gaussian distribution with a mean *gamma* and a standard deviation *delta*.

### Layer-by-layer activation strategy

In this part, the Layer-by-layer activation strategy (LAS) is introduced to avoid prem-ature convergence. The LAS is inspired by the structure of society of wild chimpanzee population, where weak individuals will be abnegated due to limited living spaces. In LAS, candidates of each particle will be assigned to compare with each layer of memories. The best m positions will be retained and the rest will be abandoned, where m is the depth of the memory space. Specifically, the memories of the ith particle in the tth iteration are calculated with Eq 3:

$$\begin{array}{c} \begin{array}{c} candi\_memory=Fusion(\left[m\_p\_p{\left(i\right)}^{t},candidate\_x{\left(i\right)}^{t}\right])\\ m\_p\_p{\left(m,i\right)}^{t+1}=Sel\left(candi\_memory,m\right) \end{array} \end{array}$$ (3)

where *m*_*p*_*p*(*i*)^*t*+1^ is the memory list of the ith particle in the t+1th iteration, *candidate*_*x*(*i*)^*t*^ is candidate positions calculated by Eq (3), *Fusion*() is a fusion method that combines all the input data into an array for easy calculation, *Sel*(*candi*_*memory*, *m*) is a selection functions to select best *m* positions from *candi*_*memory*, *m* is the depth of the memory space.

### The complete process of DMBBPSO

**Fig 1:**
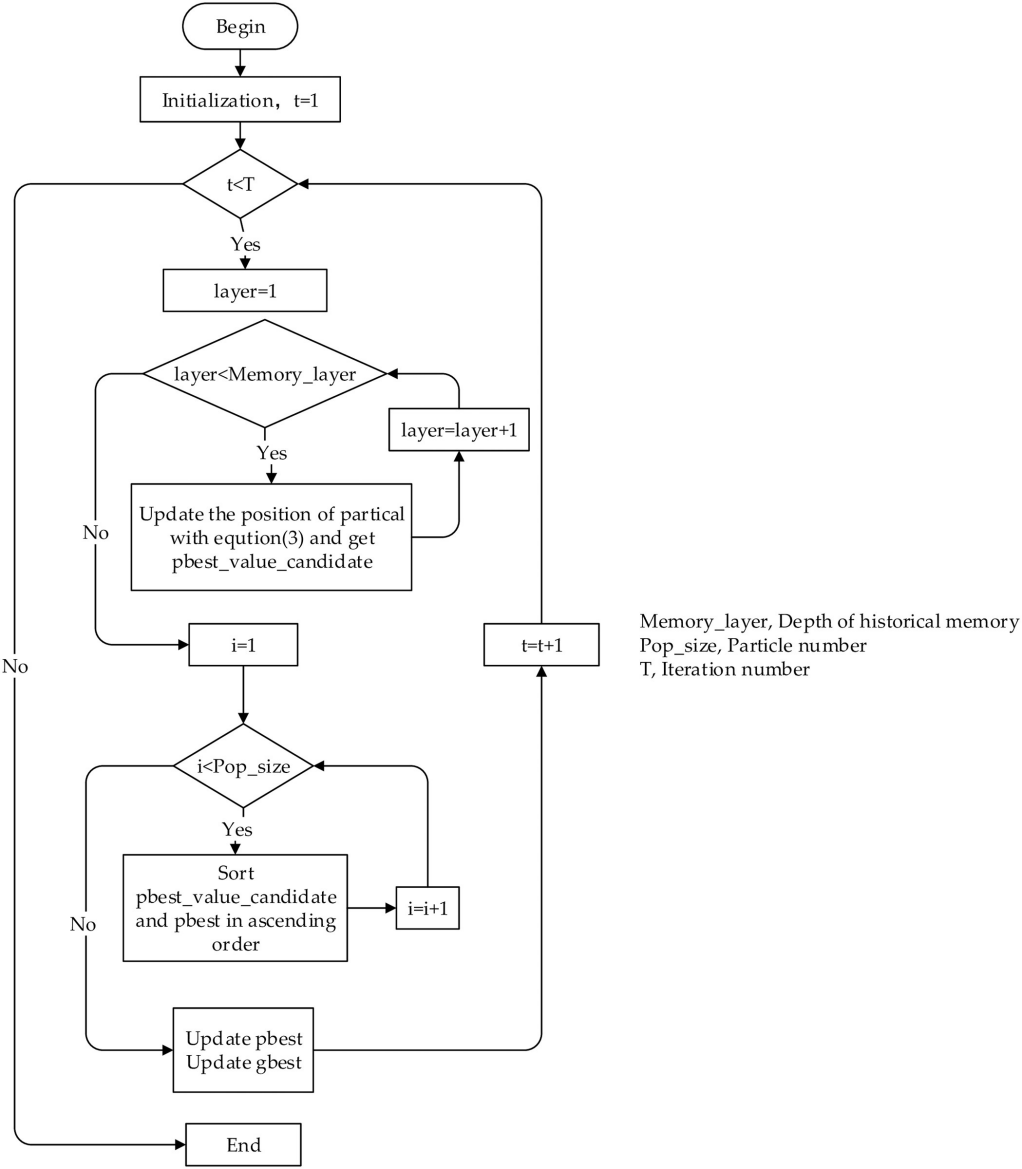
The flowchart of DMBBPSO.

The complete process of the DMBBPSO is presented in this session. After generating multiple layers of memory, the layer-by-layer activation strategy is applied to each particle. In the iteration, the M-layer memory of each particle will be stored by MMSM, and then the LAS is performed to preserve the stronger values and eliminate the worse ones. In order to demonstrate the algorithm more distinctly, the pseudo-code of the DMBBPSO is given in Algorithm 1, the flowchart is shown in Fig 1.

Here we will discuss the time complexity of DMBBPSO. In each iteration, two layers of memory of each particle are computed once with the global best particle respectively, so the computation is 2n. Each particle will update the candidate position based on computation results. After that, the global best particle will update its position according to the updated positions of the all particles. The process of position-updating only requires comparison and no computation. So this part will not generate any computation. To sum up, the time complexity of DMBBPSO is *o*(*n*).

## Results

### Problem statement

Single-objective optimization problem aims to select the optimal solution from all alternatives of a problem, which is still a very significant problem for research. It can be applied to practical problems in various fields, such as engineering optimization problems and scientific applications, which are usually computationally expensive and complex.

**Algorithm 1** Dynamic Particle Grouping

**Require**: Maximum number of iterations, *T*

**Require**: Fitness function, *F*

**Require**: Search Space, *R*

**Require**: Dimension of the function, *D*

**Require**: Number of particles, *n*

**Require**: Particle swarm, *X* = *x*_1_, *x*_2_, … *x*_*n*_

**Require**: Memory personal best position of X, *memory*_*pbest*_*position*

**Require**: Global best position, *gbest*

**Require**: Depth of historical memory, *M*

1: Initialization:

2: **for** *m* = 1 to *M* step 1 **do**

3:  Randomly generate the initial position of *x*(*i*)

4:  *memory*_*pbest*_*position*(*m*, *i*) = *x*(*i*)

5: **end for**

6: Evolution:

7: *t* = 1

8: **while** *t* < *T* **do**

9:  **for** *m*=1 to *M* step 1 **do**

10:   **for** *i*=1 to *n* step 1 **do**

11:    Calculate *candidate*_*x*(*i*) of each particle with Eq 2

12:   **end for**

13:  **end for**

14:  **for** *i*=1 to *n* step 1 **do**

15:   Merge *memory*_*pbest*_*position*(*i*) and *candidate*_*x*(*i*)

16:   Update *memory*_*pbest*_*position*(*i*) by Eq 3

17:  **end for**

18:  Update *gbest*

19:  *t* = *t* + 1

20: **end while**

21: Output *gbest*

In practical applications, optimization algorithms usually do not arrive at the theoretically optimal solution. Therefore, to compare the performance of different algorithms, we use we use the eventual error (EE) as the judgment criterion, where EE is defined as |*final* *gbest* *value* − *Theoretically* *optimal*|. A smaller EE means that the algorithm has stronger optimization capabilities.

### Experimental methods

In this section, simulation tests are evaluated to verify the performance of DMBBPSO. To enhance the persuasiveness of the experiments, cutting-edge and publicly available benchmark functions, CEC2017, are selected in experiments. CEC2017 includes 29 functions with novel features, for example, composing test functions by extraction of features dimension-wise from among functions, graded level of linkages, rotated trap functions, which is designed for real parameter single objective optimization. The FisBBPSO, PBBPSO, TBBPSO, ETBBPSO are selected to be control groups. Details of CEC2017BF are pretended in Table 1. Details of experimental parameters are shown in Table 2. Details of the control group are shown in Table 3.

**Table 1:** Experimental functions, the CEC 2017 benchmark functions, the search range for each function is [-100,100].

Types	Function	Theoretically Optimal
Unimodal Functions	*f*_1_=Shifted and Rotated Bent Cigar Function	100
	*f*_2_=Shifted and Rotated Zakharov Function	200
Simple Multimodal Functions	*f*_3_=Shifted and Rotated Rosenbrock’s Function	300
	*f*_4_=Shifted and Rotated Rastrigin’s Function	400
	*f*_5_=Shifted and Rotated Expanded Scaffer’s f6 Function	500
	*f*_6_=Shifted and Rotated Lunacek Bi_Rastrigin Function	600
	*f*_7_=Shifted and Rotated Non-Continuous Rastrigin’s Function	700
	*f*_8_=Shifted and Rotated Levy Function	800
	*f*_9_=Shifted and Rotated Schwefel’s Function	900
Hybrid Functions	*f*_10_=Hybrid Function 1 (N = 3)	1000
	*f*_11_=Hybrid Function 2 (N = 3)	1100
	*f*_12_=Hybrid Function 3 (N = 3)	1200
	*f*_13_=Hybrid Function 4 (N = 4)	1300
	*f*_14_=Hybrid Function 5 (N = 4)	1400
	*f*_15_=Hybrid Function 6 (N = 4)	1500
	*f*_16_=Hybrid Function 6 (N = 5)	1600
	*f*_17_=Hybrid Function 6 (N = 5)	1700
	*f*_18_=Hybrid Function 6 (N = 5)	1800
	*f*_19_=Hybrid Function 6 (N = 6)	1900
Composition Functions	*f*_20_=Composition Function 1 (N = 3)	2000
	*f*_21_=Composition Function 2 (N = 3)	2100
	*f*_22_=Composition Function 3 (N = 4)	2200
	*f*_23_=Composition Function 4 (N = 4)	2300
	*f*_24_=Composition Function 5 (N = 5)	2400
	*f*_25_=Composition Function 6 (N = 5)	2500
	*f*_26_=Composition Function 7 (N = 6)	2600
	*f*_27_=Composition Function 8 (N = 6)	2700
	*f*_28_=Composition Function 9 (N = 3)	2800
	*f*_29_=Composition Function 10 (N = 3)	2900
Search Range: [-100,100]		

**Table 2:** The details of the test algorithm.

Parameters name	value
Population size	100
Independent Runs	37
Search Range	1.00E+04
Max iteration	10000

**Table 3:** The details of the control group.

Algorithm	Delivery Year	Reference
FisBBPSO	2019	[30]
PBBPSO	2017	[31]
TBBPSO	2022	[32]
ETBBPSO	2022	[33]

### Experimental results and discussion

The numerical results are shown in Tables 4 and 5. In *f*_2_, *f*_3_, *f*_4_, *f*_5_, *f*_8_, *f*_10_, *f*_11_, *f*_12_, *f*_14_, *f*_16_, *f*_17_, *f*_18_, *f*_19_, *f*_20_, *f*_21_, *f*_22_, *f*_23_, *f*_25_, *f*_26_, *f*_27_, *f*_28_, the DMBBPSO gains the first rank. In *f*_7_, *f*_13_, *f*_29_, the DMBBPSO gains the second rank. In *f*_1_, *f*_9_, *f*_19_, *f*_24_, the DMBBPSO gains the third rank. In *f*_6_ and *f*_15_, the DMBBPSO gains the fifth rank, these results suggest that in the face of valley-shaped function, which is tending to produce multiple local optima, the search capabilities of DMBBPSO prone to be limited. Specifically, numerical analyses are presented for critical discussions. In this paper, when we compare two algorithms means we compare their EEs.

- In *f*_1_, DMBBPSO gains the third rank, 28.09% worse than PBBPSO, the best algorithm.
- In *f*_2_, DMBBPSO gains the first rank, 100.00% better than TBBPSO, the second-best algorithm.
- In *f*_3_, DMBBPSO gains the first rank, 9.72% better than TBBPSO, the second-best algorithm.
- In *f*_4_, DMBBPSO gains the first rank, 10.91% better than FisBBPSO, the second-best algorithm.
- In *f*_5_, DMBBPSO gains the first rank, 1.19% better than ETBBPSO, the second-best algorithm.
- In *f*_6_, DMBBPSO gains the fifth rank, 9.45% worse than TBBPSO, the best algorithm.
- In *f*_7_, DMBBPSO gains the second rank, 1.97% worse than ETBBPSO, the best algorithm.
- In *f*_8_, DMBBPSO gains the first rank, 8.63% better than ETBBPSO, the second-best algorithm.
- In *f*_9_, DMBBPSO gains the third rank, 5.59% worse than PBBPSO, the best algorithm.
- In *f*_10_, DMBBPSO gains the first rank, 13.40% better than FisBBPSO, the second-best algorithm.
- In *f*_11_, DMBBPSO gains the first rank, 89.85% better than PBBPSO, the second-best algorithm.
- In *f*_12_, DMBBPSO gains the first rank, 30.20% better than TBBPSO, the second-best algorithm.
- In *f*_13_, DMBBPSO gains the second rank, 44.20% worse than TBBPSO, the second-best algorithm.
- In *f*_14_, DMBBPSO gains the first rank, 48.05% better than PBBPSO, the second-best algorithm.
- In *f*_15_, DMBBPSO gains the fifth rank, 100.05% worse than PBBPSO, the best algorithm.
- In *f*_16_, DMBBPSO gains the first rank, 19.147% better than TBBPSO, the best algorithm.
- In *f*_17_, DMBBPSO gains the first rank, 2.74% better than FisBBPSO, the second-best algorithm.
- In *f*_18_, DMBBPSO gains the first rank,46.54% better than TBBPSO, the second-best algorithm.
- In *f*_19_, DMBBPSO gains the first rank, 51.10% better than PBBPSO, the second-best algorithm.
- In *f*_20_, DMBBPSO gains the first rank, 14.29% better than ETBBPSO, the second-best algorithm.
- In *f*_21_, DMBBPSO gains the first rank, 4.67% better than TBBPSO, the second-best algorithm.
- In *f*_22_, DMBBPSO gains the first rank, 18.82% better than ETBBPSO, the second-best algorithm.
- In *f*_23_, DMBBPSO gains the first rank, 1.22% better than TBBPSO, the second-best algorithm.
- In *f*_24_, DMBBPSO gains the third rank, 3.35% worse than PBBPSO, the best algorithm.
- In *f*_25_, DMBBPSO gains the first rank, 0.02% better than TBBPSO, the second-best algorithm.
- In *f*_26_, DMBBPSO gains the first rank, 3.26% better than PBBPSO, the second-best algorithm.
- In *f*_27_, four algorithms give the same results.
- In *f*_28_, four algorithms give the same results.
- In *f*_29_, DMBBPSO gains the second rank, 0.54% worse than ETBBPSO, the best algorithm.

**Table 4:** Experimental results and average rank. EEs of DMBBPSO, FisBBPSO, PBBPSO, TBBPSO and ET-BBPSO. Mean EE is the average EE of 37 experiments, Std is the standard deviation of 37 EEs, Rank is the rank competition results based on EEs. *f*_1_–*f*_15_.

Function Number	Data Type	DMBBPSO	FisBBPSO	PBBPSO	TBBPSO	ETBBPSO
*f*_1_	Mean EE	2.061E+04	2.174E+04	1.609E+04	2.403E+04	1.639E+04
	Std	2.528E+04	2.596E+04	2.367E+04	3.460E+04	2.367E+04
	Rank	3	4	1	5	2
*f*_2_	Mean EE	4.696E+96	1.241E+134	1.209E+137	7.324E+118	2.963E+128
	Std	1.531E+97	6.908E+134	7.351E+137	3.439E+119	1.802E+129
	Rank	1	4	5	2	3
*f*_3_	Mean EE	1.737E+06	2.995E+06	3.683E+06	1.924E+06	2.864E+06
	Std	9.362E+05	2.325E+06	2.993E+06	7.351E+05	2.389E+06
	Rank	1	4	5	2	3
*f*_4_	Mean EE	1.360E+02	1.527E+02	1.800E+02	1.737E+02	1.716E+02
	Std	3.257E+01	5.018E+01	6.134E+01	5.786E+01	5.101E+01
	Rank	1	2	5	4	3
*f*_5_	Mean EE	8.823E+02	1.024E+03	9.414E+02	9.294E+02	8.929E+02
	Std	2.057E+02	1.466E+02	1.446E+02	1.631E+02	1.582E+02
	Rank	1	5	4	3	2
*f*_6_	Mean EE	4.081E+01	3.988E+01	3.960E+01	3.729E+01	3.872E+01
	Std	9.078E+00	7.429E+00	7.868E+00	7.132E+00	9.221E+00
	Rank	5	4	3	1	2
*f*_7_	Mean EE	9.171E+02	9.579E+02	9.258E+02	9.669E+02	8.994E+02
	Std	2.235E+02	1.555E+02	1.728E+02	1.926E+02	1.635E+02
	Rank	2	4	3	5	1
*f*_8_	Mean EE	7.985E+02	9.591E+02	9.428E+02	9.370E+02	8.740E+02
	Std	1.406E+02	1.574E+02	1.674E+02	1.840E+02	1.563E+02
	Rank	1	5	4	3	2
*f*_9_	Mean EE	3.676E+04	3.677E+04	3.482E+04	3.592E+04	3.832E+04
	Std	6.538E+03	7.240E+03	1.742E+04	1.090E+04	7.871E+03
	Rank	3	4	1	2	5
*f*_10_	Mean EE	1.857E+04	2.145E+04	3.146E+04	2.571E+04	2.301E+04
	Std	7.535E+03	8.098E+03	3.570E+03	5.069E+03	9.342E+03
	Rank	1	2	5	4	3
*f*_11_	Mean EE	4.417E+02	7.161E+03	4.351E+03	5.135E+03	7.200E+03
	Std	1.969E+02	8.651E+03	4.259E+03	1.018E+04	2.465E+04
	Rank	1	4	2	3	5
*f*_12_	Mean EE	3.202E+07	4.587E+07	4.727E+07	4.967E+07	4.746E+07
	Std	1.454E+07	2.502E+07	3.105E+07	2.764E+07	2.125E+07
	Rank	1	2	3	5	4
*f*_13_	Mean EE	8.825E+03	1.169E+04	1.518E+04	6.120E+03	1.853E+04
	Std	1.111E+04	1.321E+04	1.932E+04	5.777E+03	2.730E+04
	Rank	2	3	4	1	5
*f*_14_	Mean EE	5.754E+05	1.347E+06	1.108E+06	1.341E+06	1.274E+06
	Std	3.286E+05	8.529E+05	6.696E+05	7.917E+05	7.567E+05
	Rank	1	5	2	4	3
*f*_15_	Mean EE	1.205E+04	1.041E+04	6.022E+03	7.596E+03	8.918E+03
	Std	1.572E+04	1.240E+04	7.023E+03	7.655E+03	1.159E+04
	Rank	5	4	1	2	3

**Table 5:** Experimental results and average rank. EEs of DMBBPSO, FisBBPSO, PBBPSO, TBBPSO and ET-BBPSO. Mean EE is the average EE of 37 experiments, Std is the standard deviation of 37 EEs, Rank is the rank competition results based on EEs, *f*_16_–*f*_29_.

Function Number	Data Type	DMBBPSO	FisBBPSO	PBBPSO	TBBPSO	ETBBPSO
*f*_16_	Mean EE	5.240E+03	6.566E+03	8.887E+03	6.480E+03	6.624E+03
	Std	6.981E+02	2.590E+03	2.706E+03	2.493E+03	2.414E+03
	Rank	1	3	5	2	4
*f*_17_	Mean EE	4.723E+03	4.856E+03	6.005E+03	4.955E+03	5.171E+03
	Std	7.406E+02	1.205E+03	1.513E+03	1.233E+03	9.961E+02
	Rank	1	2	5	3	4
*f*_18_	Mean EE	2.636E+06	6.789E+06	7.217E+06	4.932E+06	6.719E+06
	Std	1.177E+06	3.763E+06	5.035E+06	4.422E+06	3.899E+06
	Rank	1	4	5	2	3
*f*_19_	Mean EE	3.645E+03	7.993E+03	7.453E+03	8.500E+03	9.853E+03
	Std	5.753E+03	1.088E+04	9.692E+03	9.840E+03	1.355E+04
	Rank	1	3	2	4	5
*f*_20_	Mean EE	3.112E+03	3.914E+03	4.983E+03	3.750E+03	3.631E+03
	Std	4.908E+02	1.130E+03	1.504E+03	1.216E+03	1.141E+03
	Rank	1	4	5	3	2
*f*_21_	Mean EE	1.055E+03	1.188E+03	1.125E+03	1.106E+03	1.109E+03
	Std	1.274E+02	1.528E+02	1.697E+02	1.474E+02	1.502E+02
	Rank	1	5	4	2	3
*f*_22_	Mean EE	2.013E+04	2.480E+04	3.222E+04	2.623E+04	2.698E+04
	Std	7.371E+03	8.347E+03	4.319E+03	5.163E+03	8.592E+03
	Rank	1	2	5	3	4
*f*_23_	Mean EE	1.266E+03	1.307E+03	1.334E+03	1.281E+03	1.287E+03
	Std	9.421E+01	1.018E+02	1.484E+02	1.081E+02	1.166E+02
	Rank	1	4	5	2	3
*f*_24_	Mean EE	1.901E+03	1.936E+03	1.839E+03	1.875E+03	1.929E+03
	Std	1.561E+02	1.991E+02	1.657E+02	1.754E+02	2.645E+02
	Rank	3	5	1	2	4
*f*_25_	Mean EE	7.521E+02	7.636E+02	7.607E+02	7.523E+02	7.595E+02
	Std	5.244E+01	6.427E+01	5.656E+01	5.669E+01	6.666E+01
	Rank	1	5	4	2	3
*f*_26_	Mean EE	1.390E+04	1.462E+04	1.437E+04	1.482E+04	1.467E+04
	Std	1.786E+03	1.725E+03	1.554E+03	1.809E+03	1.580E+03
	Rank	1	3	2	5	4
*f*_27_	Mean EE	5.000E+02	5.000E+02	5.000E+02	5.000E+02	5.000E+02
	Std	3.535E-04	5.553E-04	3.064E-04	3.289E-04	5.400E-04
	Rank	1	3	5	4	2
*f*_28_	Mean EE	5.000E+02	5.000E+02	5.000E+02	5.000E+02	5.000E+02
	Std	3.393E-04	4.275E-04	3.206E-04	3.485E-04	4.873E-04
	Rank	1	4	5	3	2
*f*_29_	Mean EE	4.290E+03	4.303E+03	4.374E+03	4.425E+03	4.267E+03
	Std	668.2378204	868.1706402	796.0159241	590.8446466	796.7057972
	Rank	2	3	4	5	1
Average Rank		1.586	3.655	3.621	3.035	3.103

To perform the convergence situation across iterations, EEs in different iterations for DMBBPSO, FisBBPSO, PBBPSO, TBBPSO and ETBBPSO is shown in figures. The conver-gence curve of f1 to f29 is shown in Figs 2–30, separately. The scale on the vertical axis represents the value of EE. The scale on the horizontal axis represents iteration times, 10 on the horizontal axis represents 1,000 iterations.

**Fig 2:**
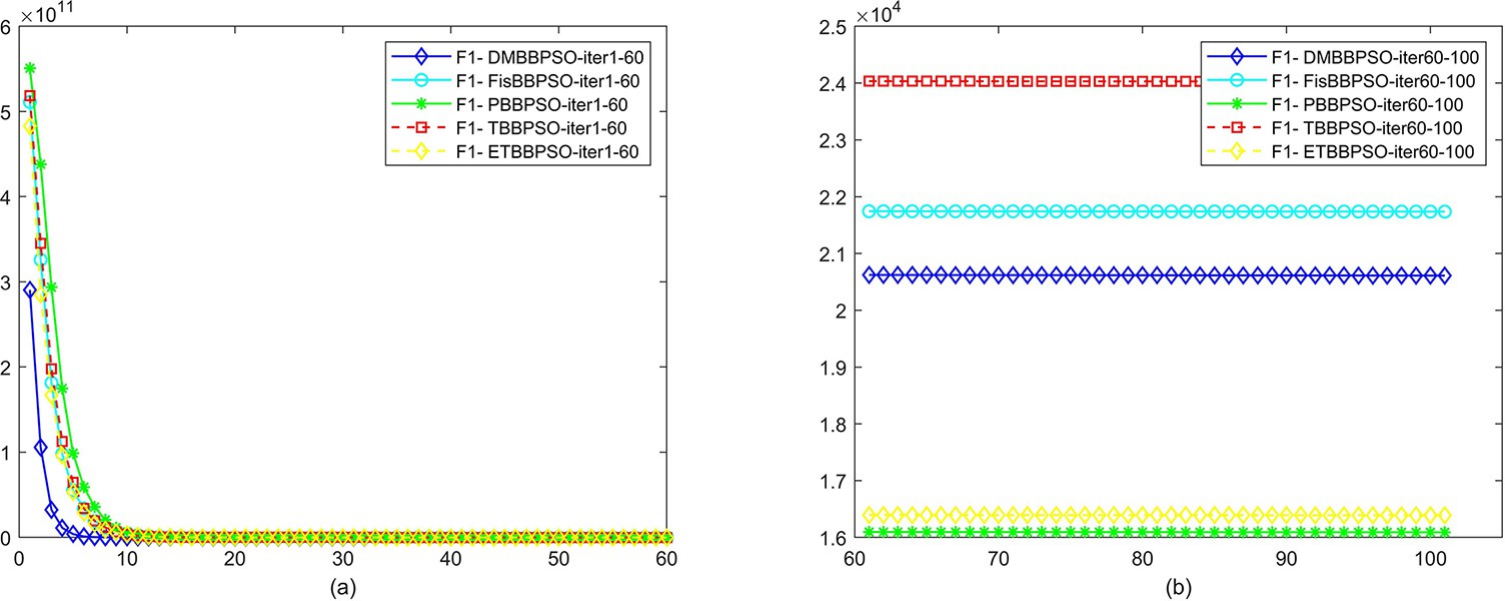
Comparison of convergence speed between DMBBPSO, FisBBPSO, PBBPSO, TBBPSO and ETBBPSO, *f*_1_. (a) iteration 0–6000, (b) iteration 6000–10000 the unit is 100 iterations.

**Fig 3:**
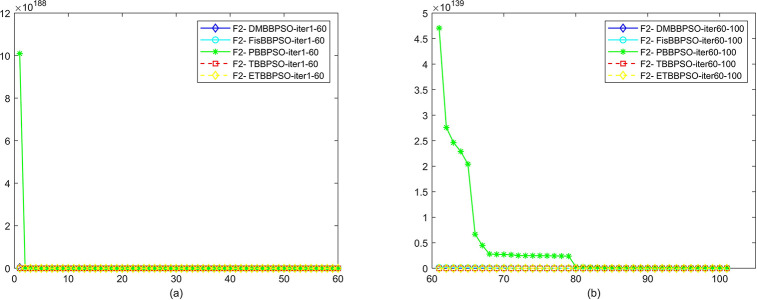
Comparison of convergence speed between DMBBPSO, FisBBPSO, PBBPSO, TBBPSO and ETBBPSO, *f*_2_. (a) iteration 0–6000, (b) iteration 6000–10000 the unit is 100 iterations.

**Fig 4:**
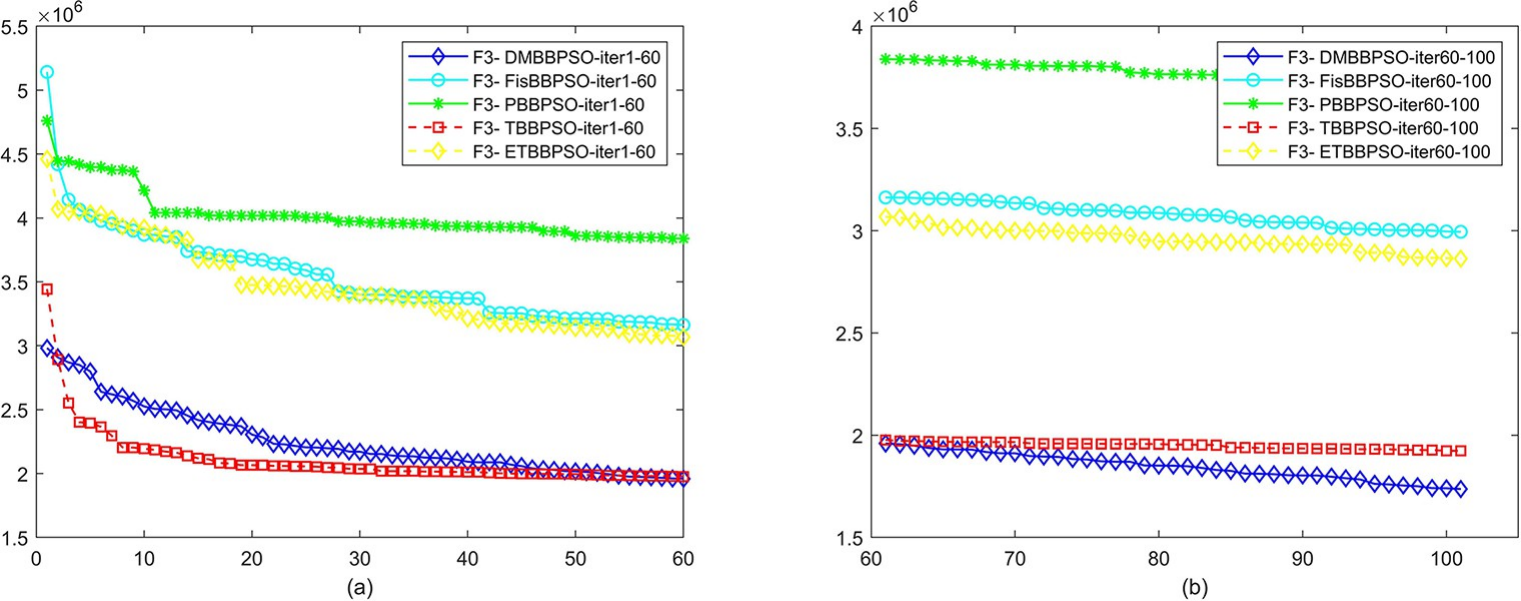
Comparison of convergence speed between DMBBPSO, FisBBPSO, PBBPSO, TBBPSO and ETBBPSO, *f*_3_. (a) iteration 0–6000, (b) iteration 6000–10000 the unit is 100 iterations.

**Fig 5:**
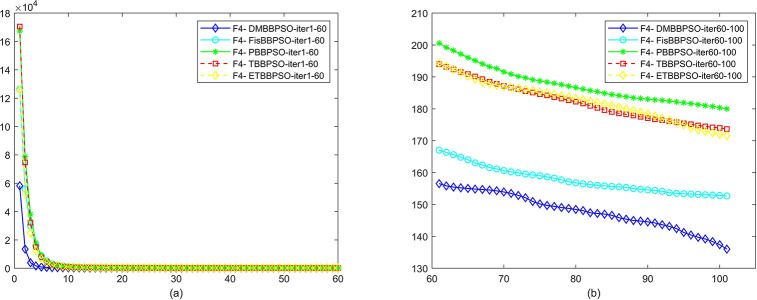
Comparison of convergence speed between DMBBPSO, FisBBPSO, PBBPSO, TBBPSO and ETBBPSO, *f*_4_. (a) iteration 0–6000, (b) iteration 6000–10000 the unit is 100 iterations.

**Fig 6:**
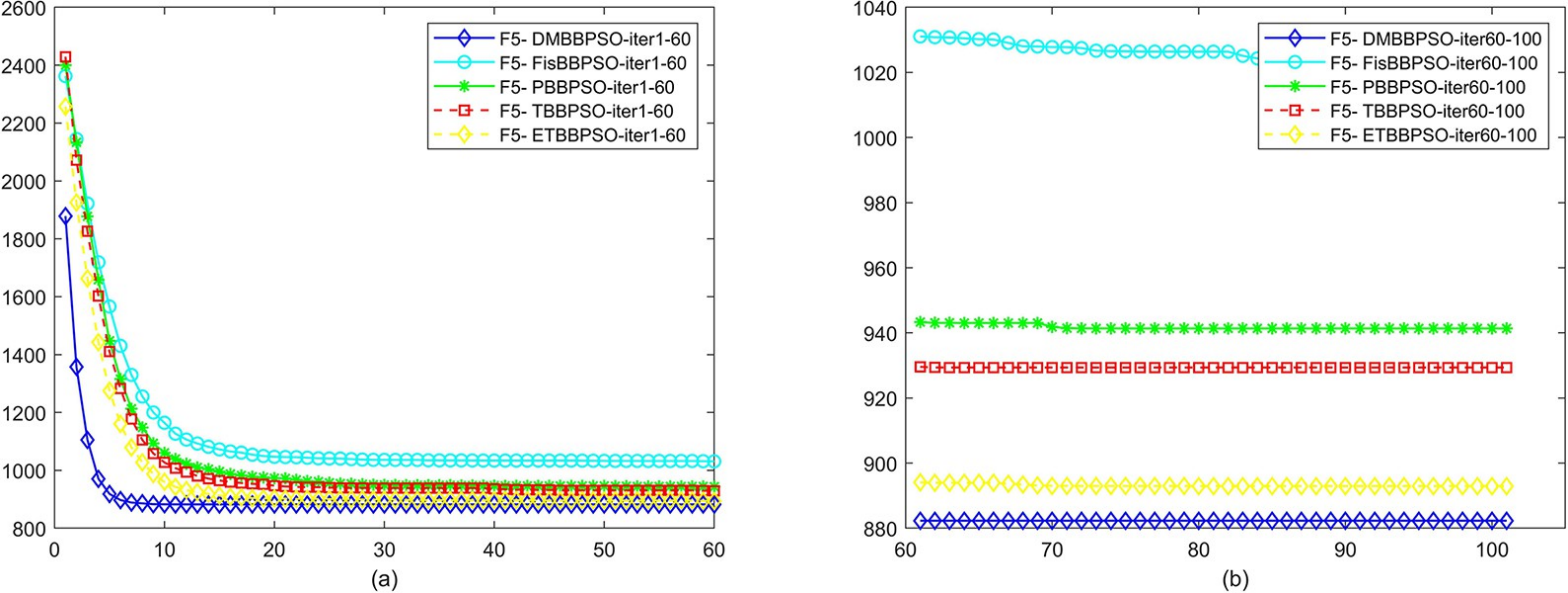
Comparison of convergence speed between DMBBPSO, FisBBPSO, PBBPSO, TBBPSO and ETBBPSO, *f*_5_. (a) iteration 0–6000, (b) iteration 6000–10000 the unit is 100 iterations.

**Fig 7:**
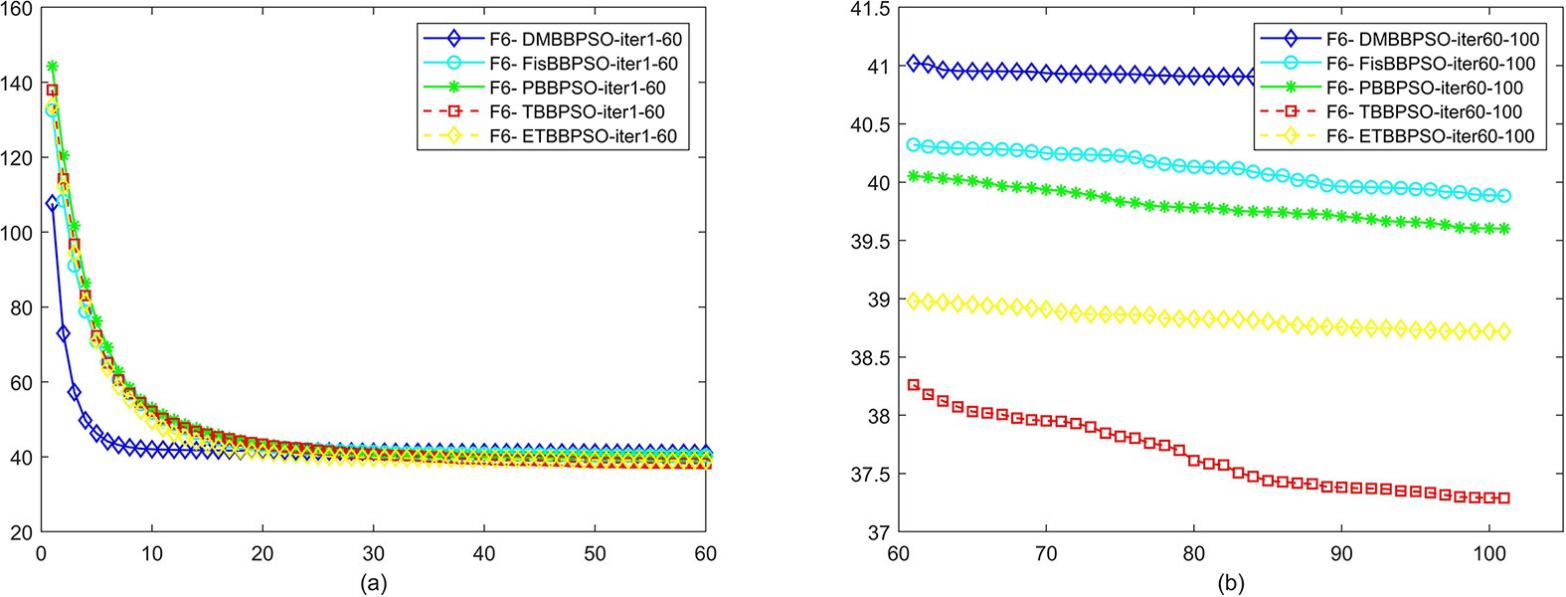
Comparison of convergence speed between DMBBPSO, FisBBPSO, PBBPSO, TBBPSO and ETBBPSO, *f*_6_. (a) iteration 0–6000, (b) iteration 6000–10000 the unit is 100 iterations.

**Fig 8:**
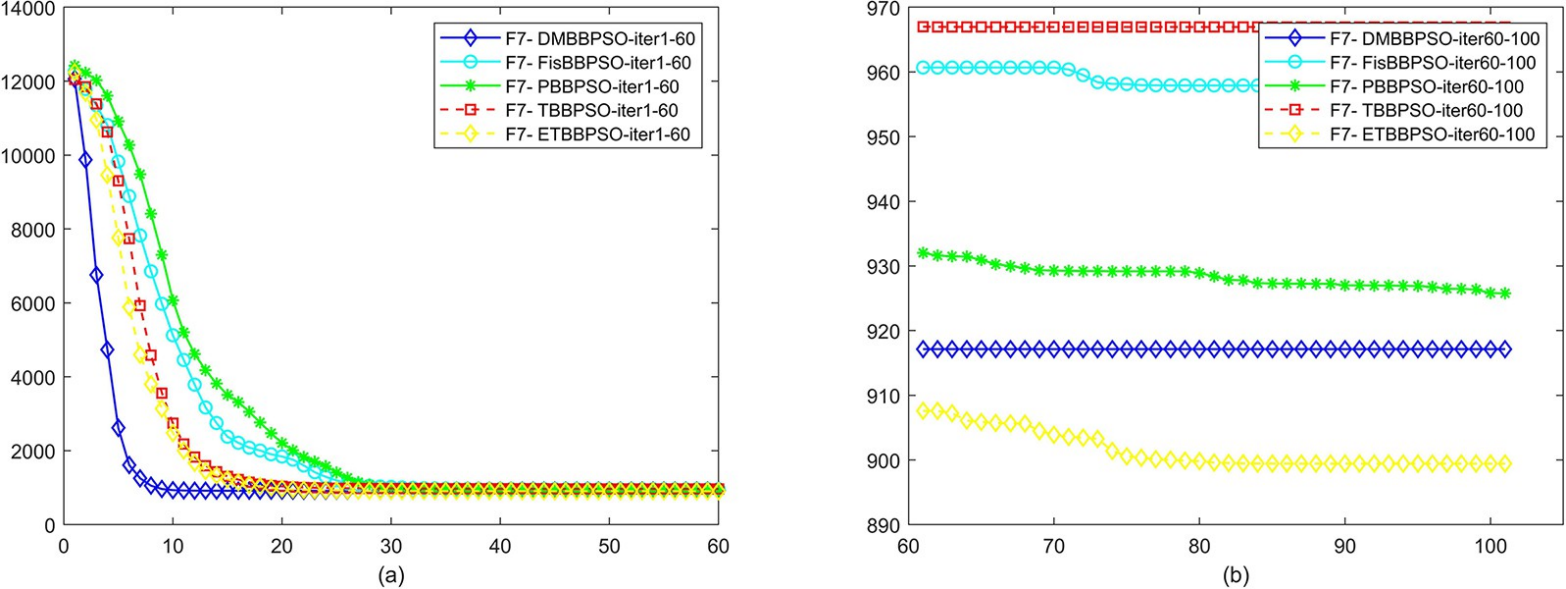
Comparison of convergence speed between DMBBPSO, FisBBPSO, PBBPSO, TBBPSO and ETBBPSO, *f*_7_. (a) iteration 0–6000, (b) iteration 6000–10000 the unit is 100 iterations.

**Fig 9:**
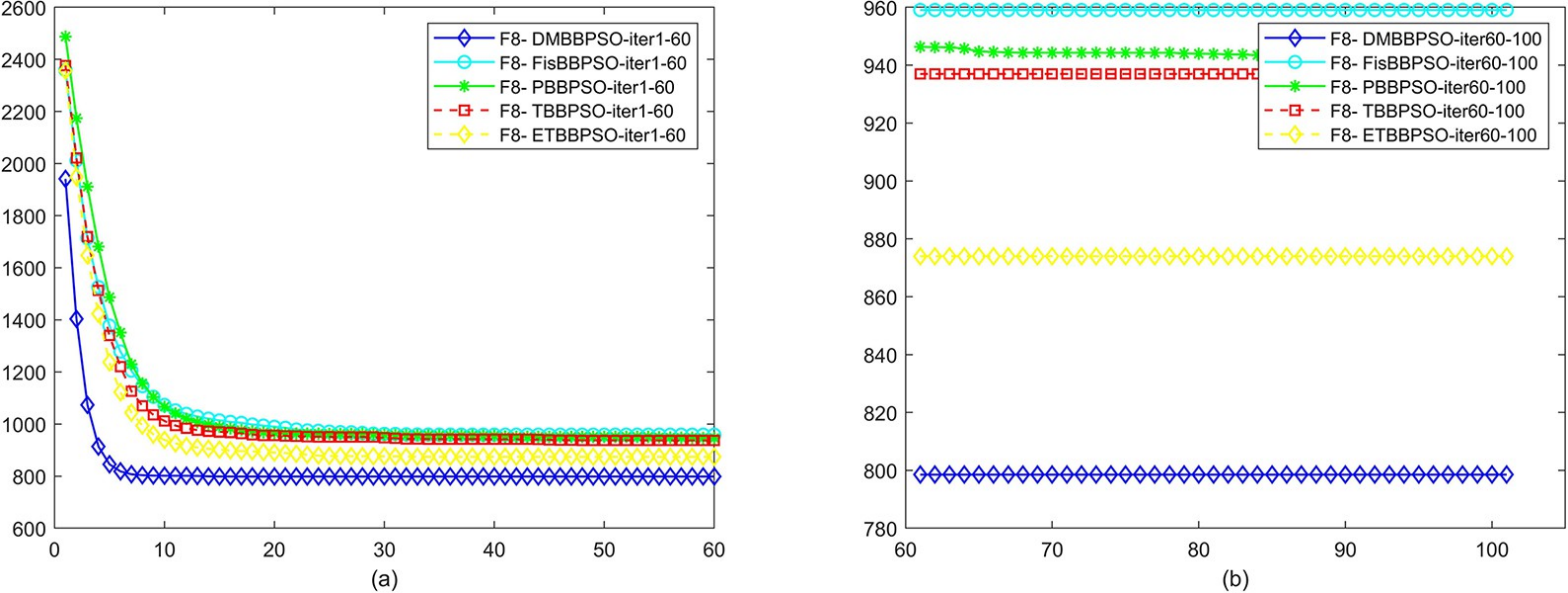
Comparison of convergence speed between DMBBPSO, FisBBPSO, PBBPSO, TBBPSO and ETBBPSO, *f*_8_. (a) iteration 0–6000, (b) iteration 6000–10000 the unit is 100 iterations.

**Fig 10:**
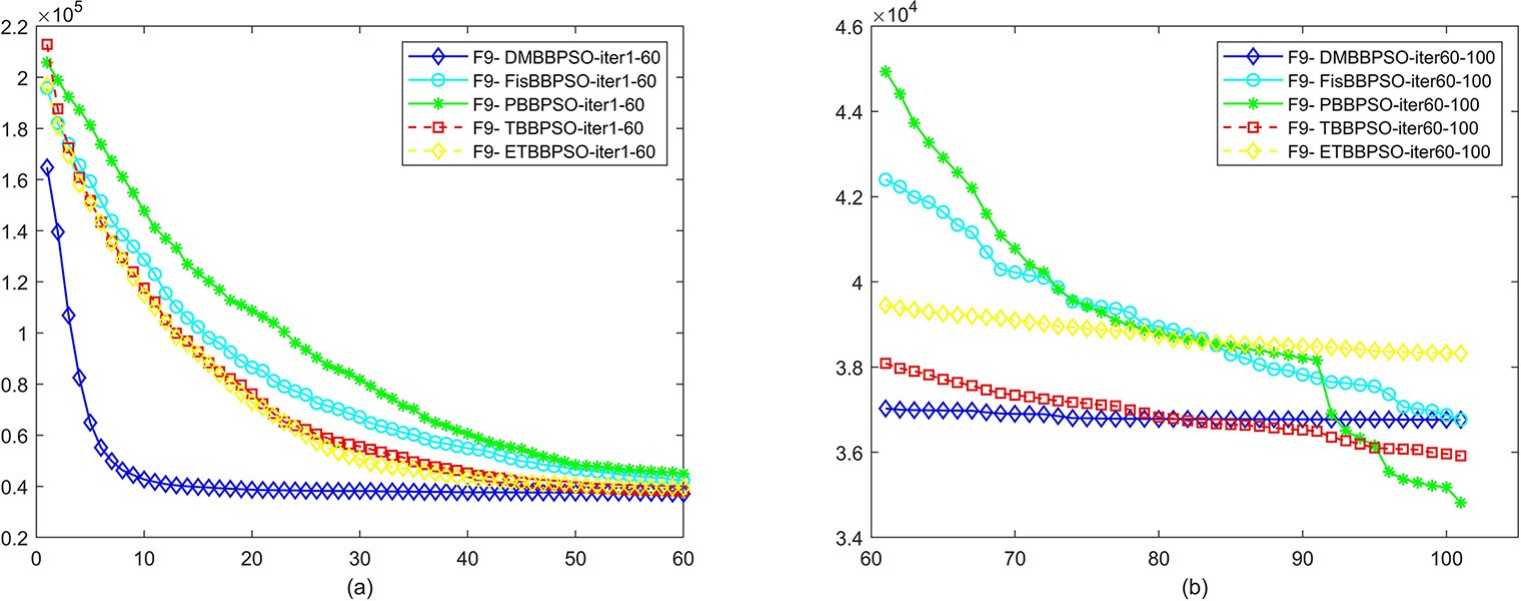
Comparison of convergence speed between DMBBPSO, FisBBPSO, PBBPSO, TBBPSO and ETBBPSO, *f*_9_. (a) iteration 0–6000, (b) iteration 6000–10000 the unit is 100 iterations.

**Fig 11:**
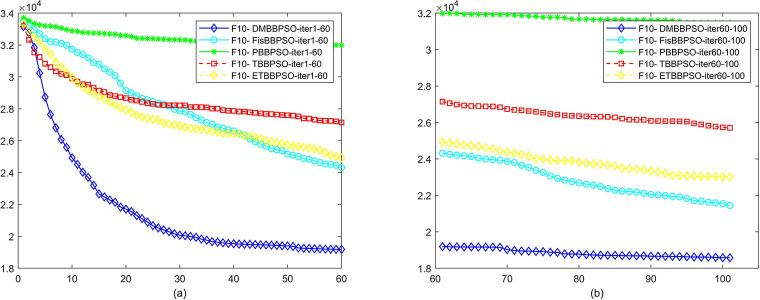
Comparison of convergence speed between DMBBPSO, FisBBPSO, PBBPSO, TBBPSO and ETBBPSO, *f*_10_. (a) iteration 0–6000, (b) iteration 6000–10000 the unit is 100 iterations.

**Fig 12:**
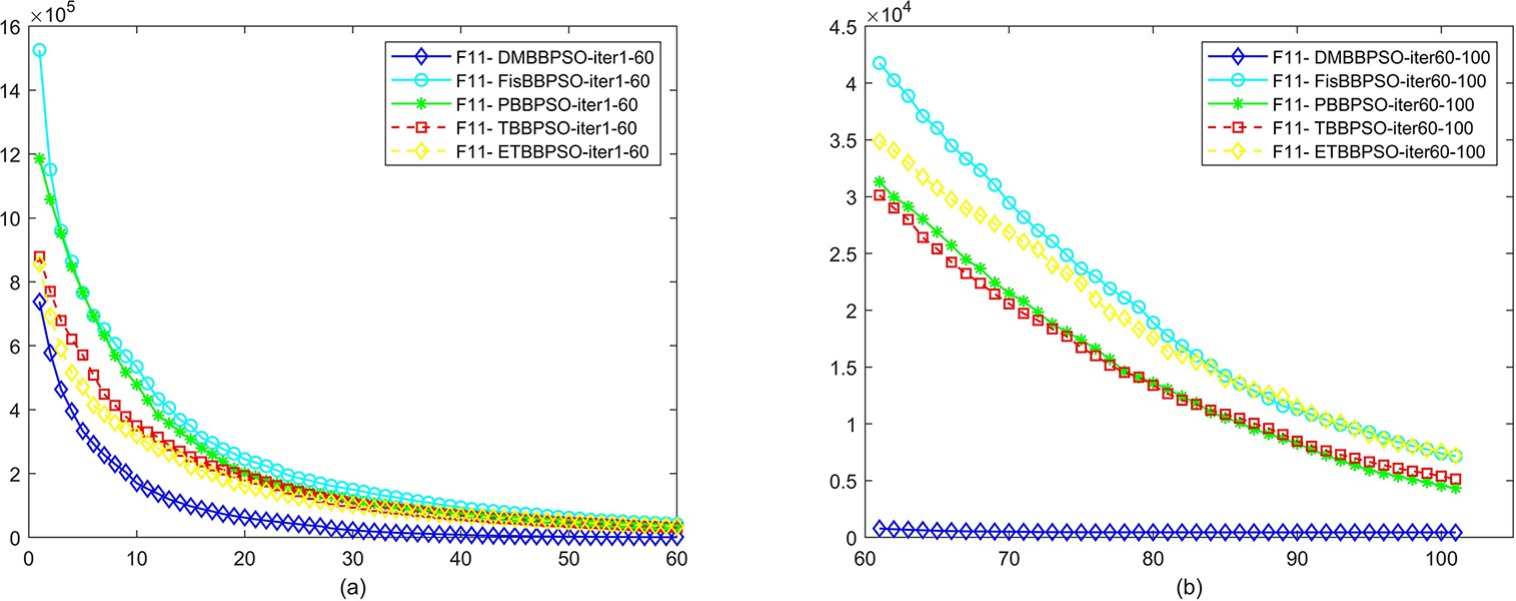
Comparison of convergence speed between DMBBPSO, FisBBPSO, PBBPSO, TBBPSO and ETBBPSO, *f*_11_. (a) iteration 0–6000, (b) iteration 6000–10000 the unit is 100 iterations.

**Fig 13:**
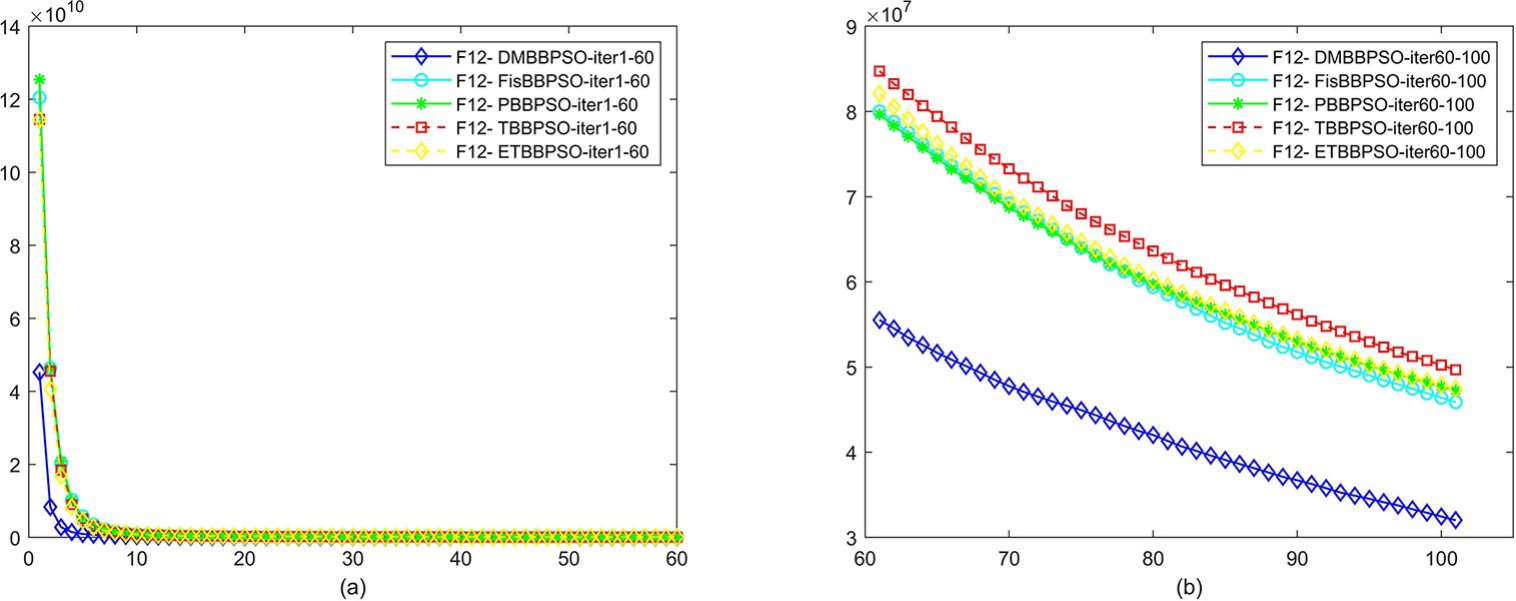
Comparison of convergence speed between DMBBPSO, FisBBPSO, PBBPSO, TBBPSO and ETBBPSO, *f*_12_. (a) iteration 0–6000, (b) iteration 6000–10000 the unit is 100 iterations.

**Fig 14:**
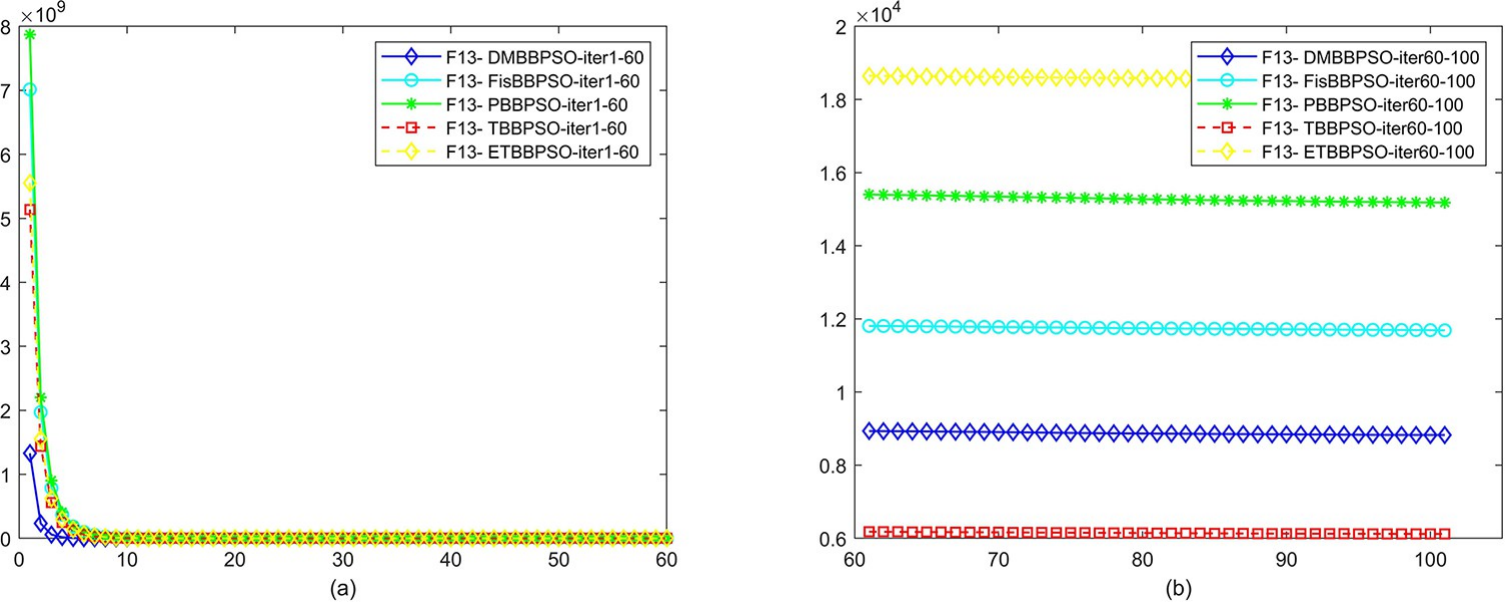
Comparison of convergence speed between DMBBPSO, FisBBPSO, PBBPSO, TBBPSO and ETBBPSO, *f*_13_. (a) iteration 0–6000, (b) iteration 6000–10000 the unit is 100 iterations.

**Fig 15:**
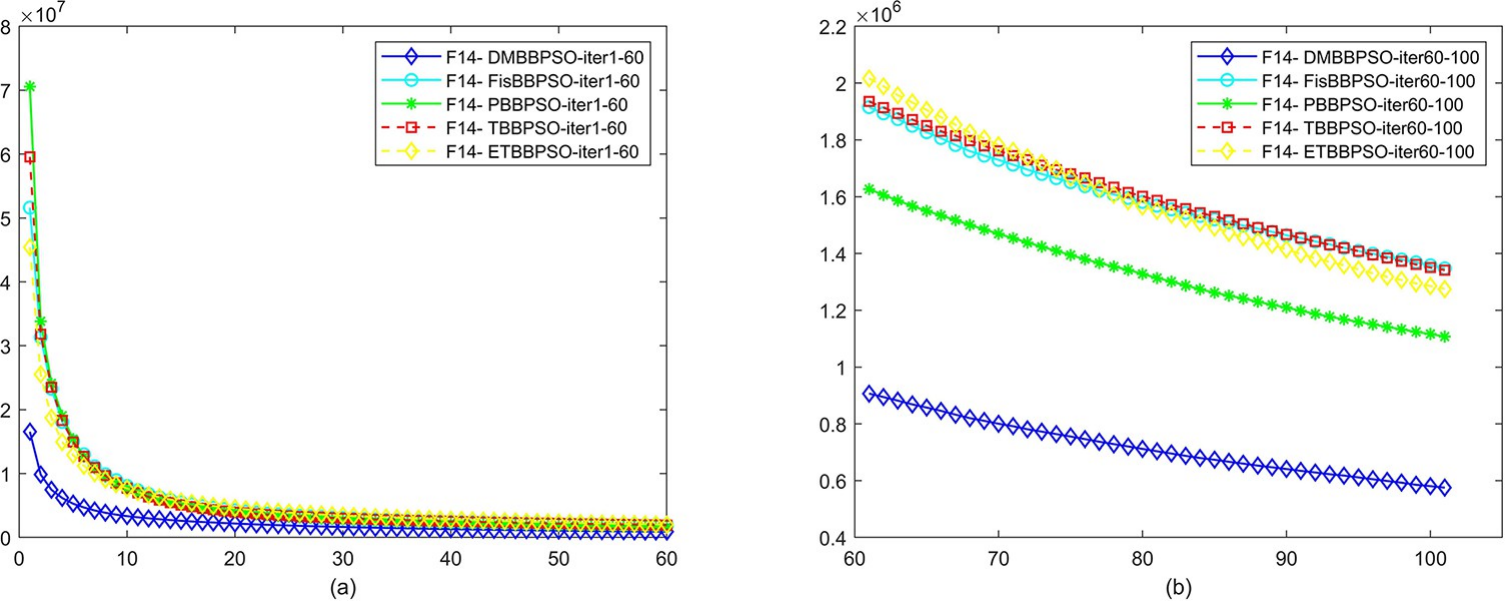
Comparison of convergence speed between DMBBPSO, FisBBPSO, PBBPSO, TBBPSO and ETBBPSO, *f*_14_. (a) iteration 0–6000, (b) iteration 6000–10000 the unit is 100 iterations.

**Fig 16:**
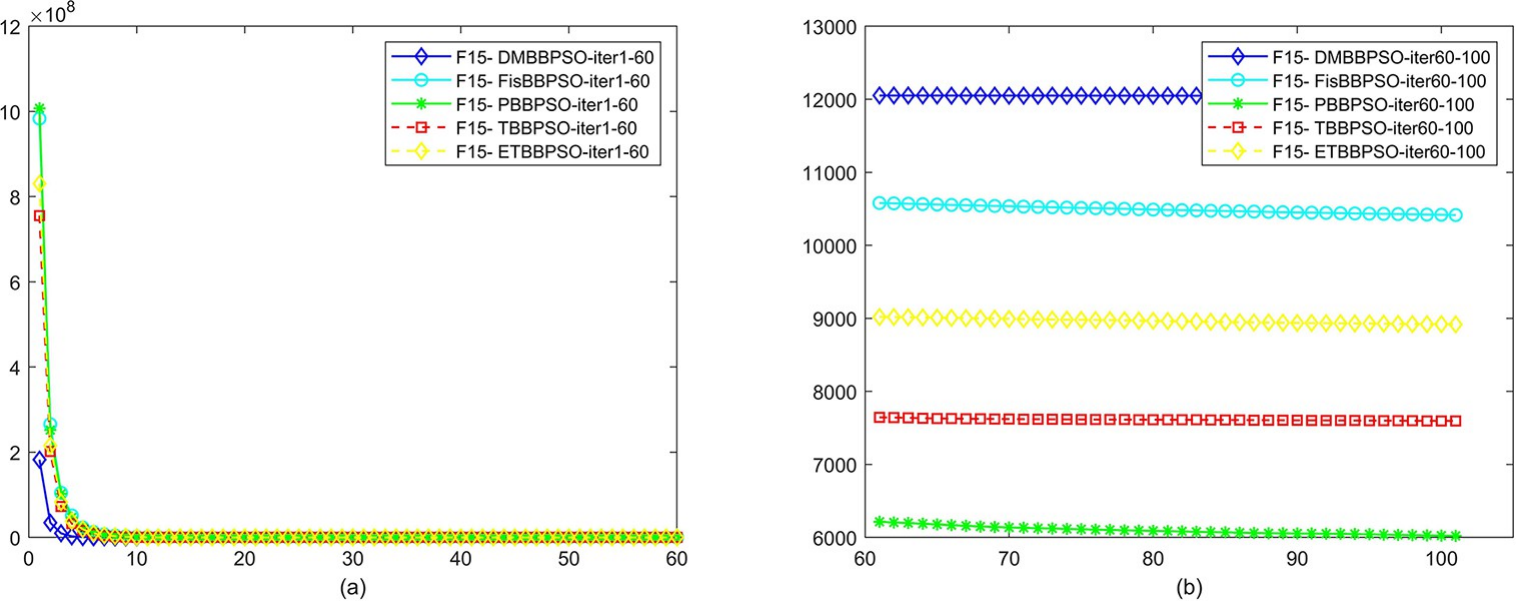
Comparison of convergence speed between DMBBPSO, FisBBPSO, PBBPSO, TBBPSO and ETBBPSO, *f*_15_. (a) iteration 0–6000, (b) iteration 6000–10000 the unit is 100 iterations.

**Fig 17:**
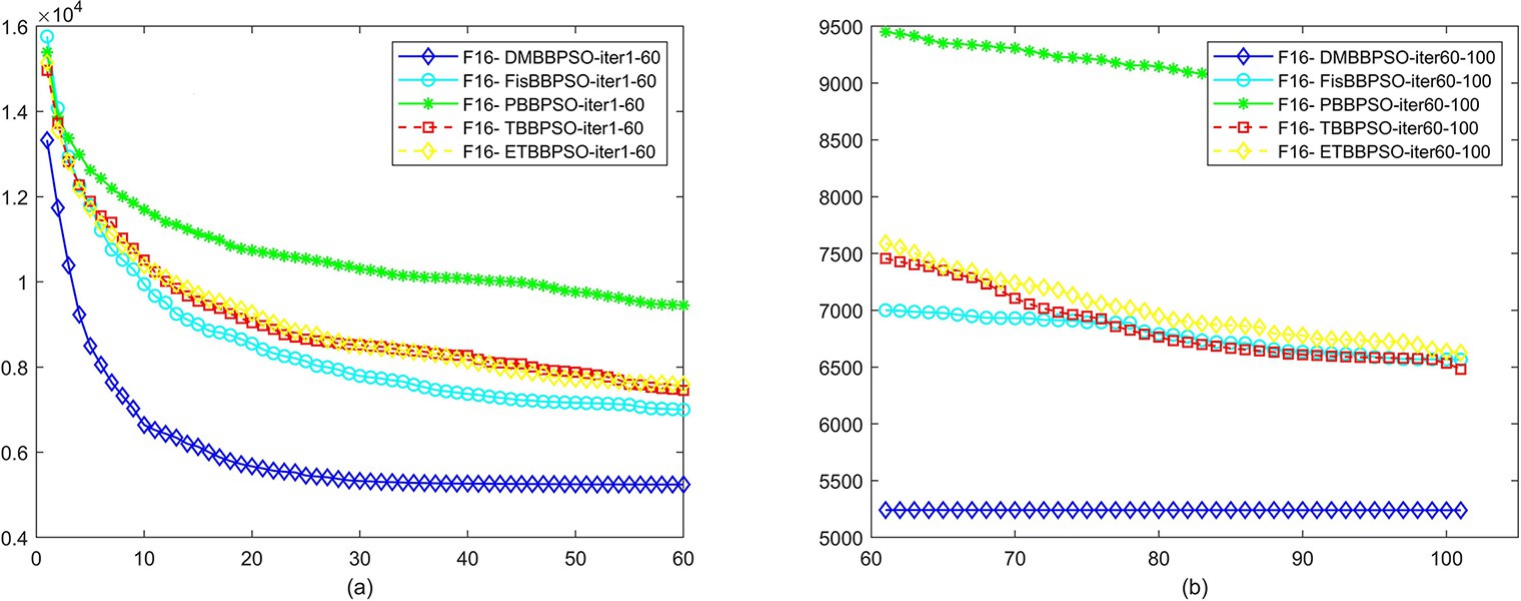
Comparison of convergence speed between DMBBPSO, FisBBPSO, PBBPSO, TBBPSO and ETBBPSO, *f*_16_. (a) iteration 0–6000, (b) iteration 6000–10000 the unit is 100 iterations.

**Fig 18:**
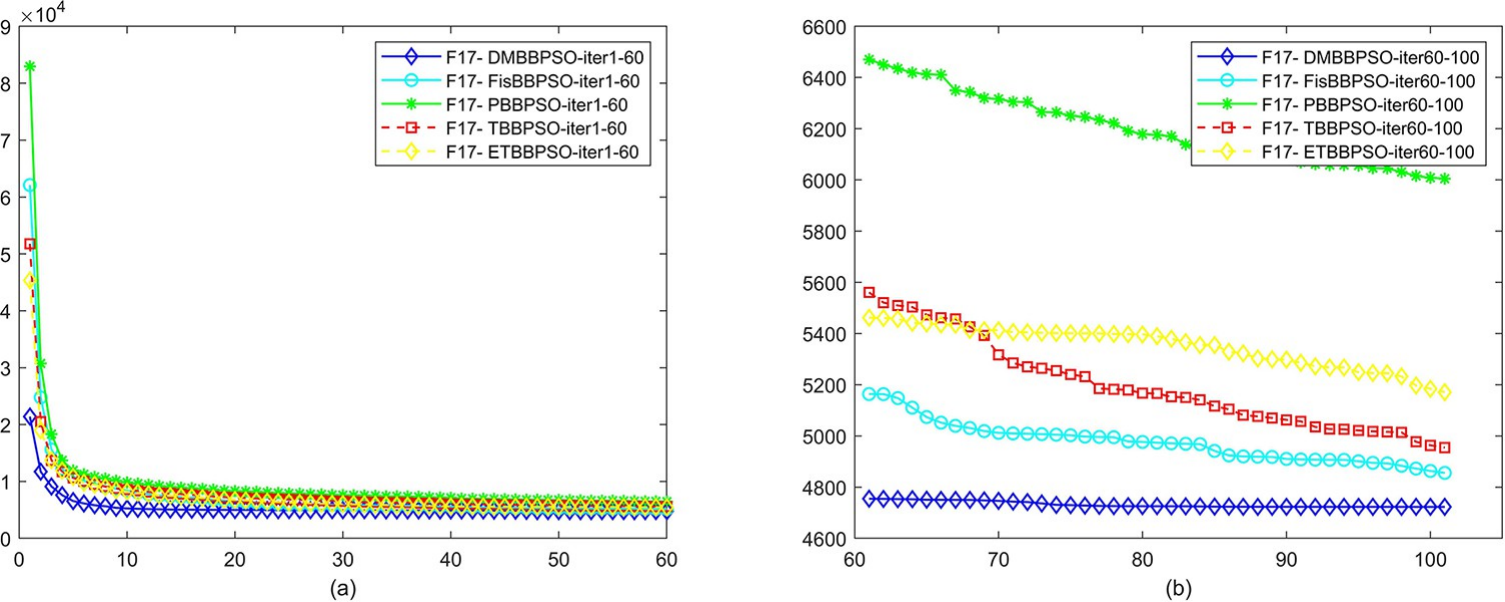
Comparison of convergence speed between DMBBPSO, FisBBPSO, PBBPSO, TBBPSO and ETBBPSO, *f*_17_. (a) iteration 0–6000, (b) iteration 6000–10000 the unit is 100 iterations.

**Fig 19:**
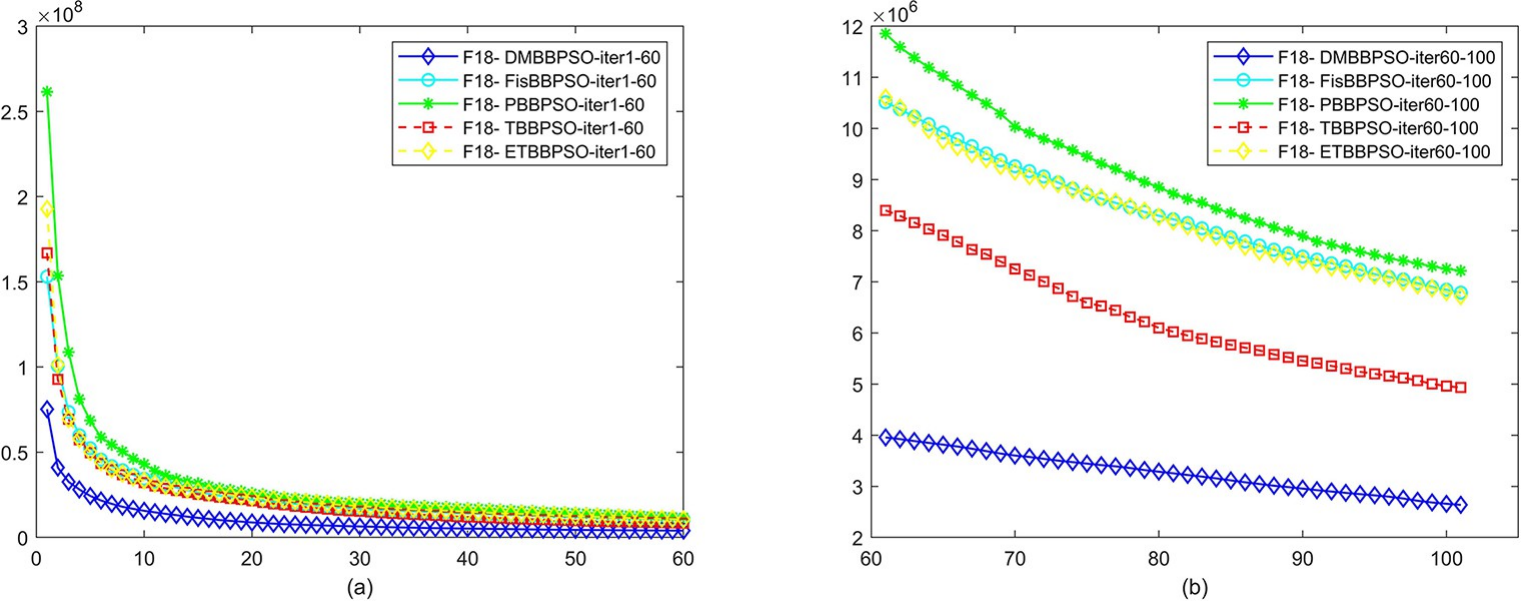
Comparison of convergence speed between DMBBPSO, FisBBPSO, PBBPSO, TBBPSO and ETBBPSO, *f*_18_. (a) iteration 0–6000, (b) iteration 6000–10000 the unit is 100 iterations.

**Fig 20:**
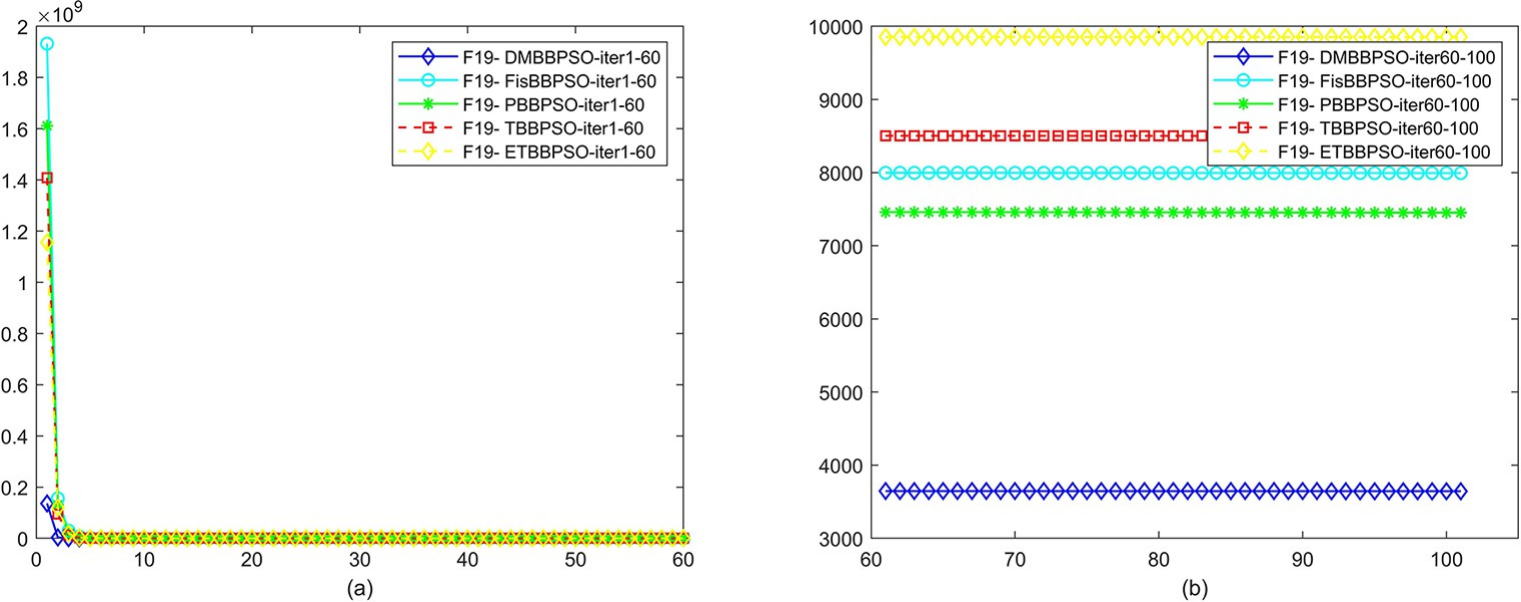
Comparison of convergence speed between DMBBPSO, FisBBPSO, PBBPSO, TBBPSO and ETBBPSO, *f*_19_. (a) iteration 0–6000, (b) iteration 6000–10000 the unit is 100 iterations.

**Fig 21:**
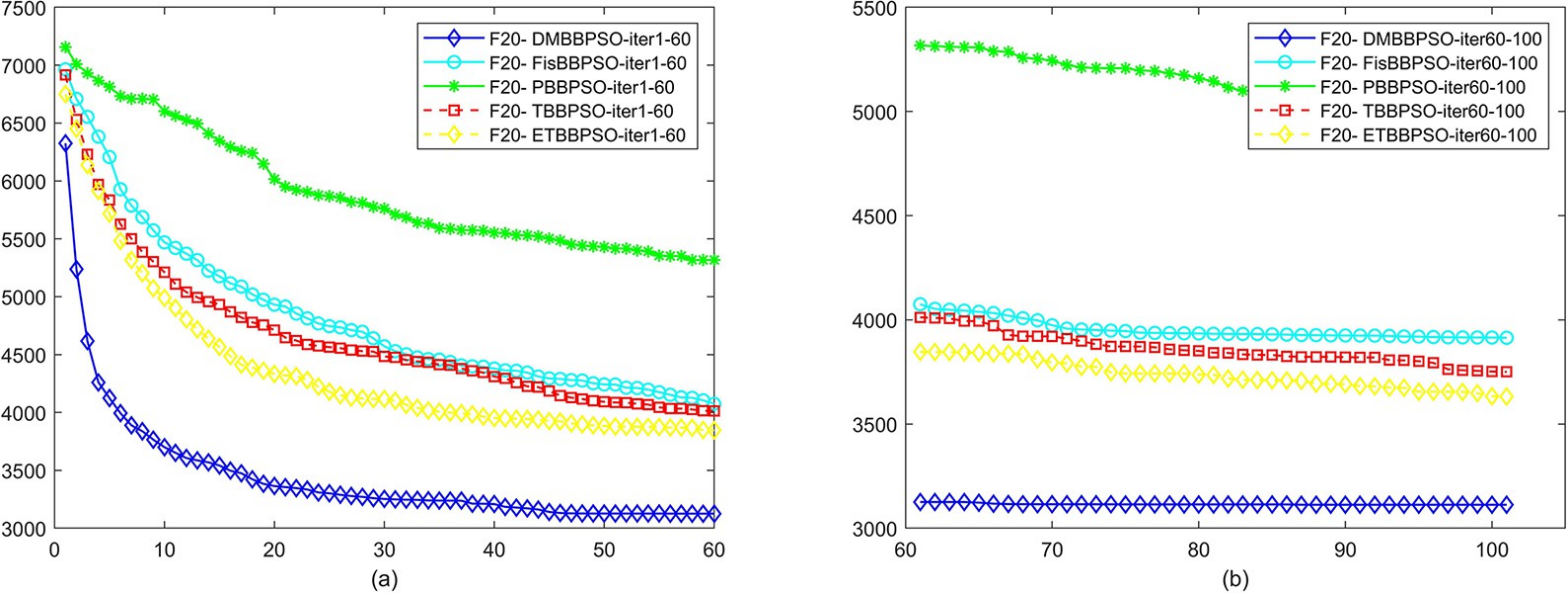
Comparison of convergence speed between DMBBPSO, FisBBPSO, PBBPSO, TBBPSO and ETBBPSO, *f*_20_. (a) iteration 0–6000, (b) iteration 6000–10000 the unit is 100 iterations.

**Fig 22:**
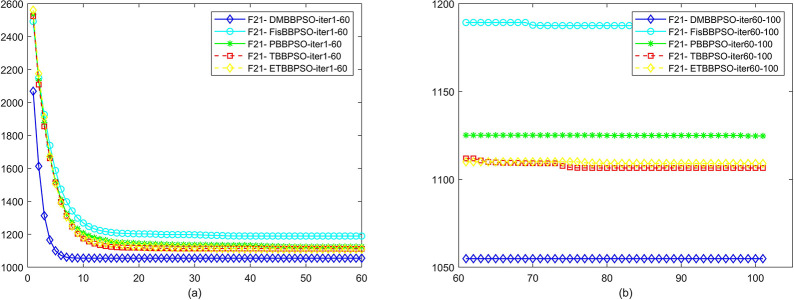
Comparison of convergence speed between DMBBPSO, FisBBPSO, PBBPSO, TBBPSO and ETBBPSO, *f*_21_. (a) iteration 0–6000, (b) iteration 6000–10000 the unit is 100 iterations.

**Fig 23:**
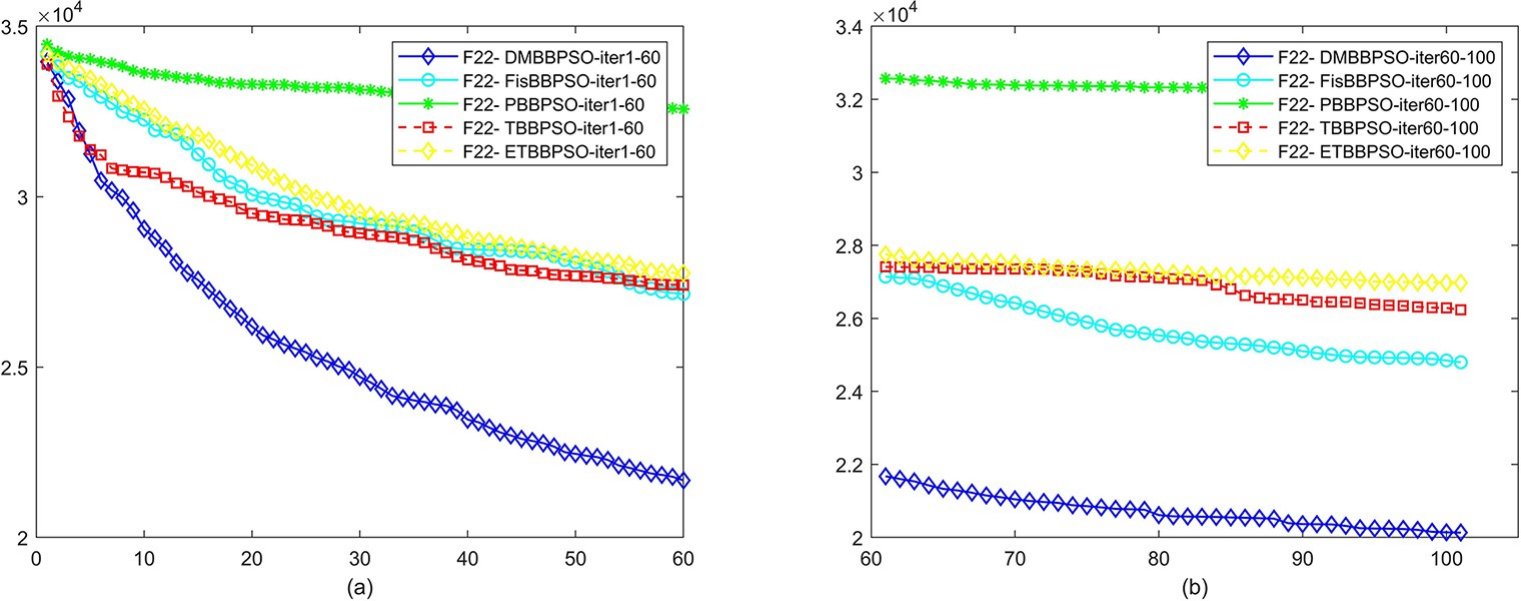
Comparison of convergence speed between DMBBPSO, FisBBPSO, PBBPSO, TBBPSO and ETBBPSO, *f*_22_. (a) iteration 0–6000, (b) iteration 6000–10000 the unit is 100 iterations.

**Fig 24:**
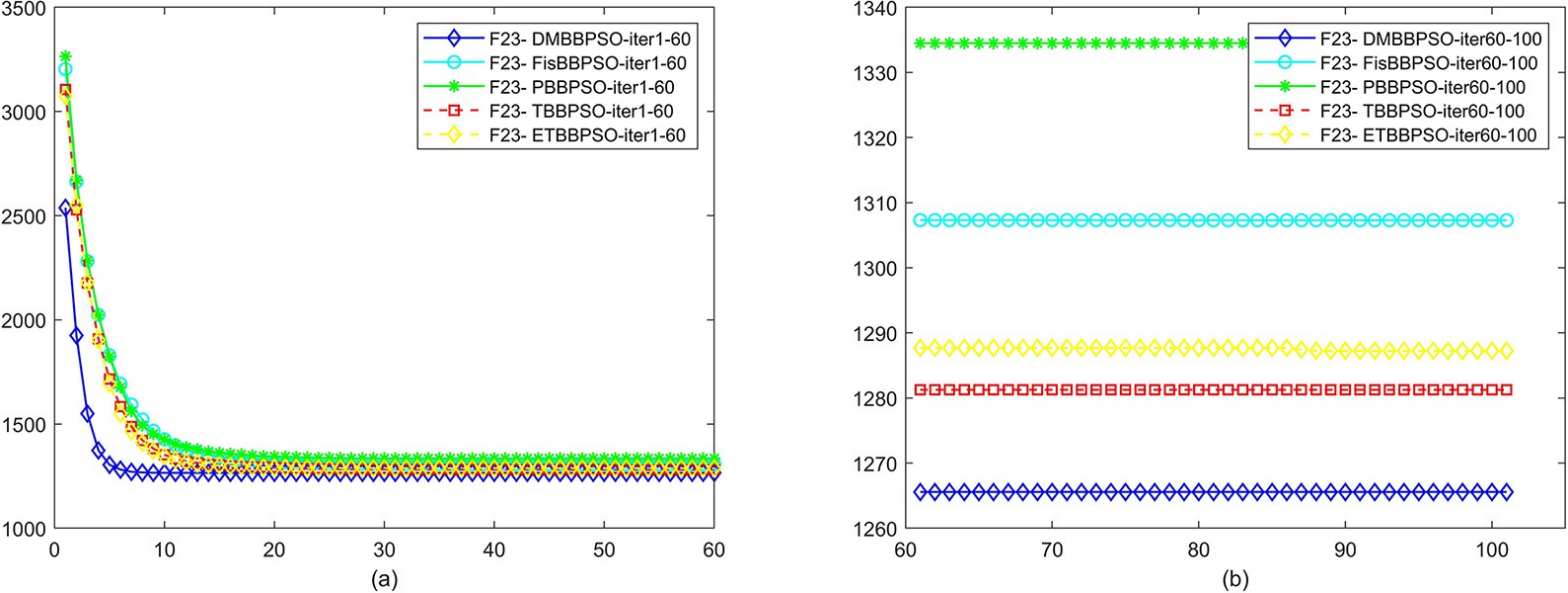
Comparison of convergence speed between DMBBPSO, FisBBPSO, PBBPSO, TBBPSO and ETBBPSO, *f*_23_. (a) iteration 0–6000, (b) iteration 6000–10000 the unit is 100 iterations.

**Fig 25:**
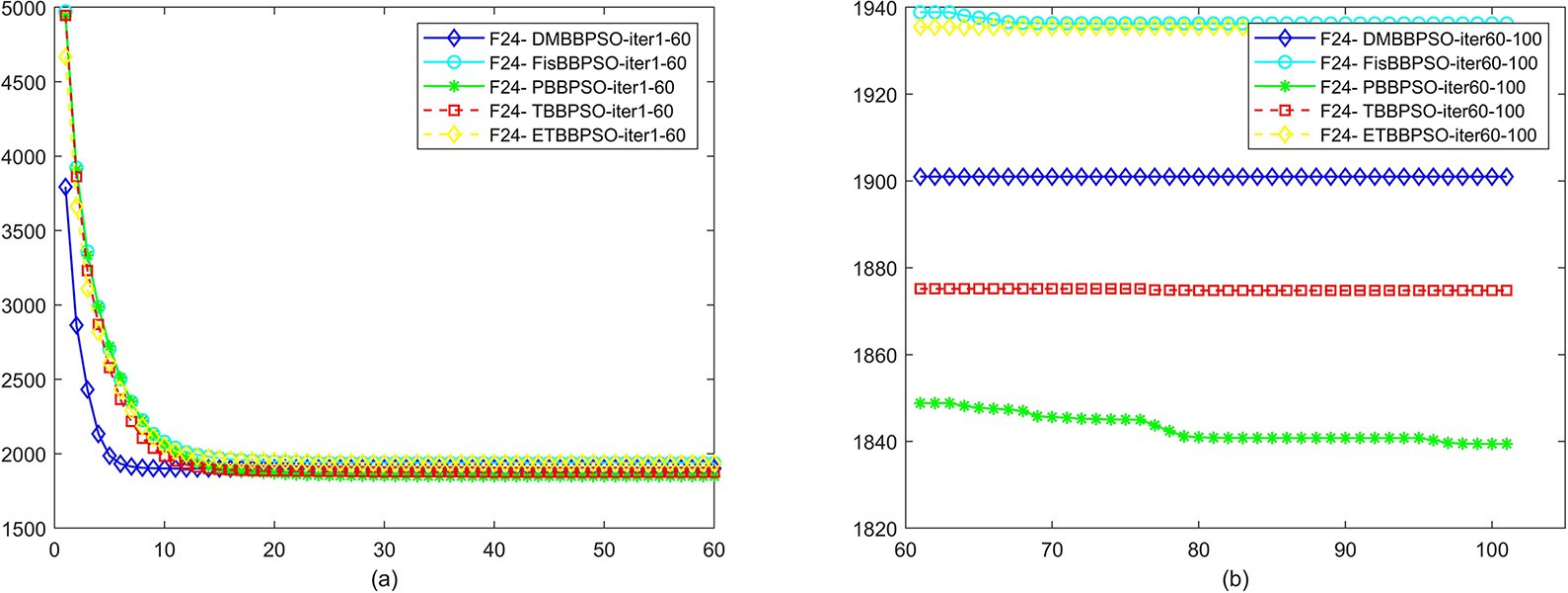
Comparison of convergence speed between DMBBPSO, FisBBPSO, PBBPSO, TBBPSO and ETBBPSO, *f*_24_. (a) iteration 0–6000, (b) iteration 6000–10000 the unit is 100 iterations.

**Fig 26:**
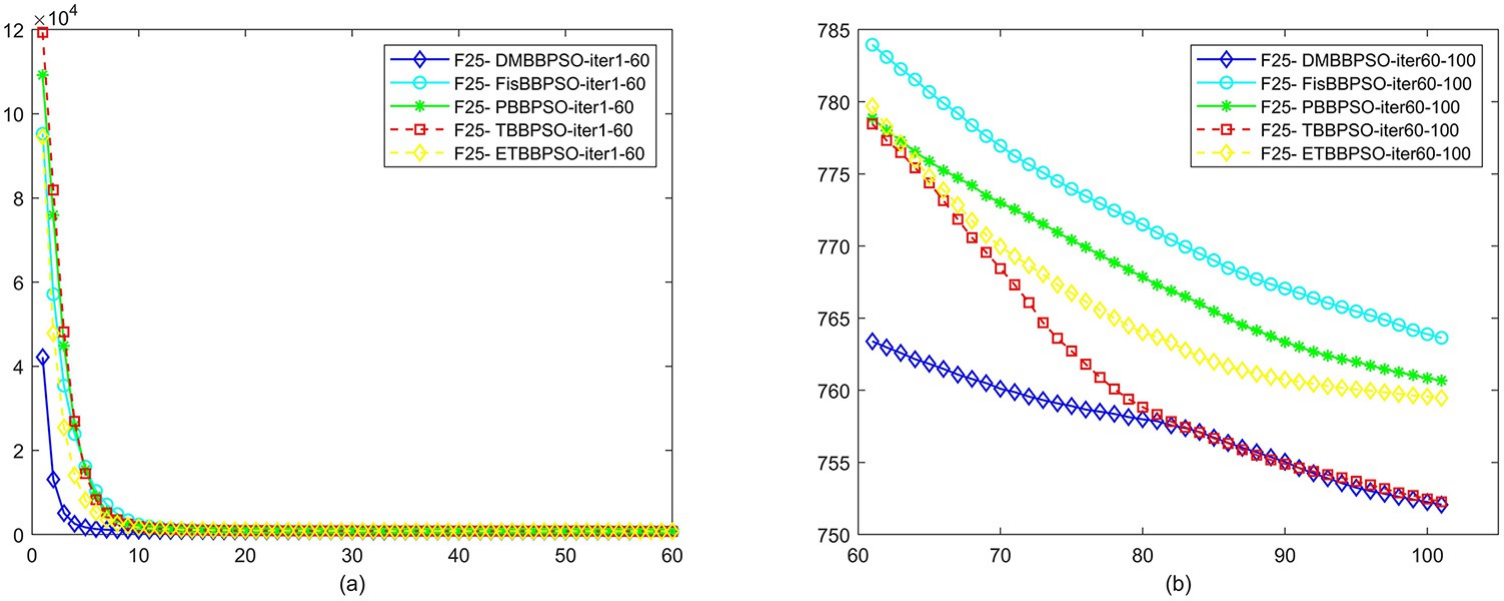
Comparison of convergence speed between DMBBPSO, FisBBPSO, PBBPSO, TBBPSO and ETBBPSO, *f*_25_. (a) iteration 0–6000, (b) iteration 6000–10000 the unit is 100 iterations.

**Fig 27:**
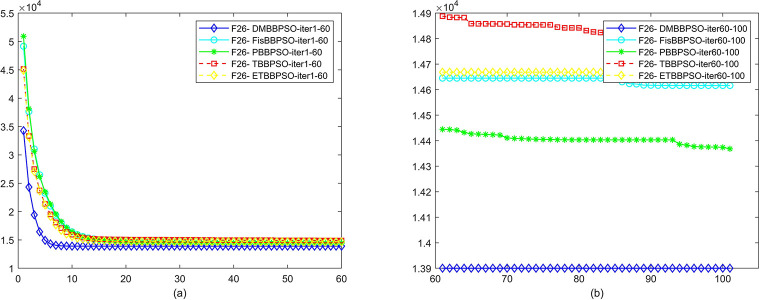
Comparison of convergence speed between DMBBPSO, FisBBPSO, PBBPSO, TBBPSO and ETBBPSO, *f*_26_. (a) iteration 0–6000, (b) iteration 6000–10000 the unit is 100 iterations.

**Fig 28:**
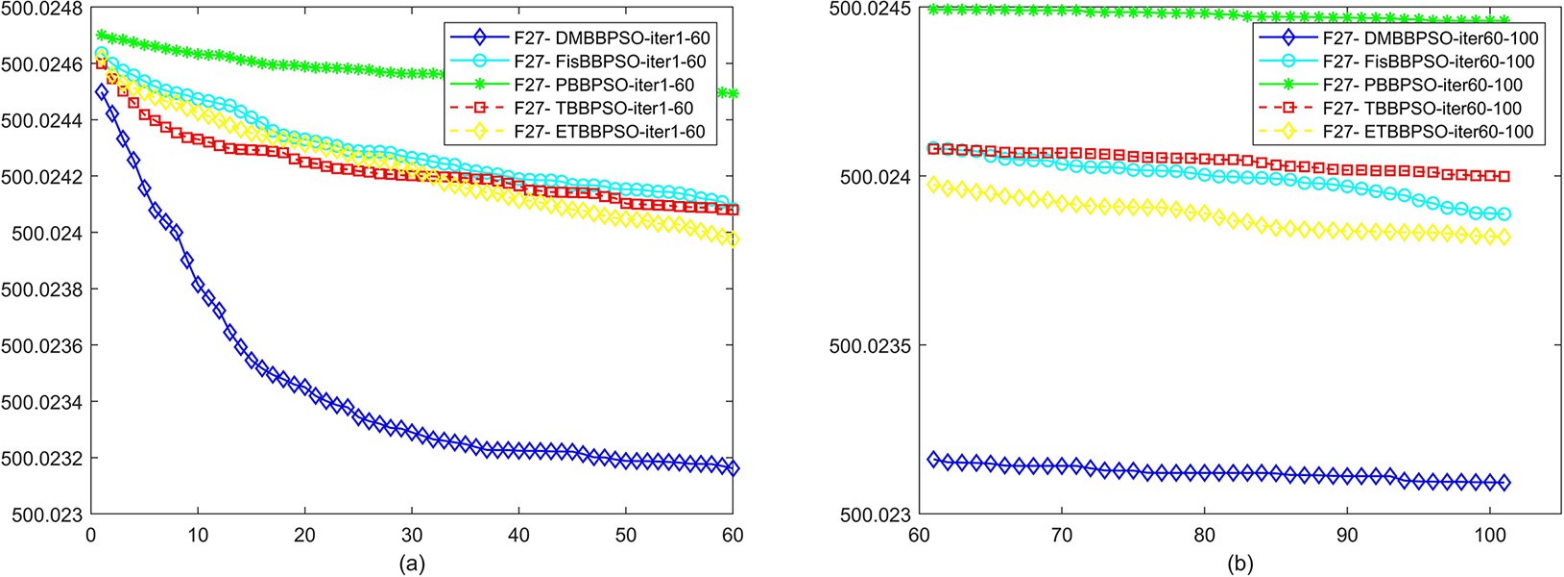
Comparison of convergence speed between DMBBPSO, FisBBPSO, PBBPSO, TBBPSO and ETBBPSO, *f*_27_. (a) iteration 0–6000, (b) iteration 6000–10000 the unit is 100 iterations.

**Fig 29:**
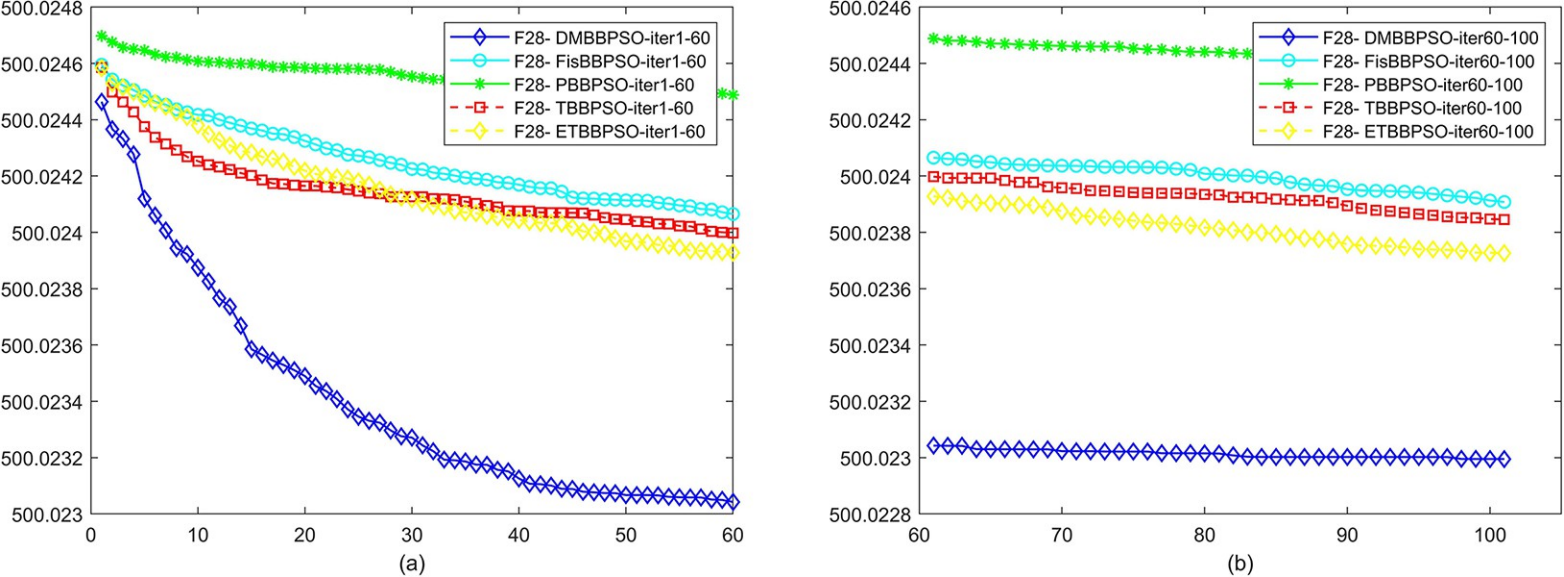
Comparison of convergence speed between DMBBPSO, FisBBPSO, PBBPSO, TBBPSO and ETBBPSO, *f*_28_. (a) iteration 0–6000, (b) iteration 6000–10000 the unit is 100 iterations.

**Fig 30:**
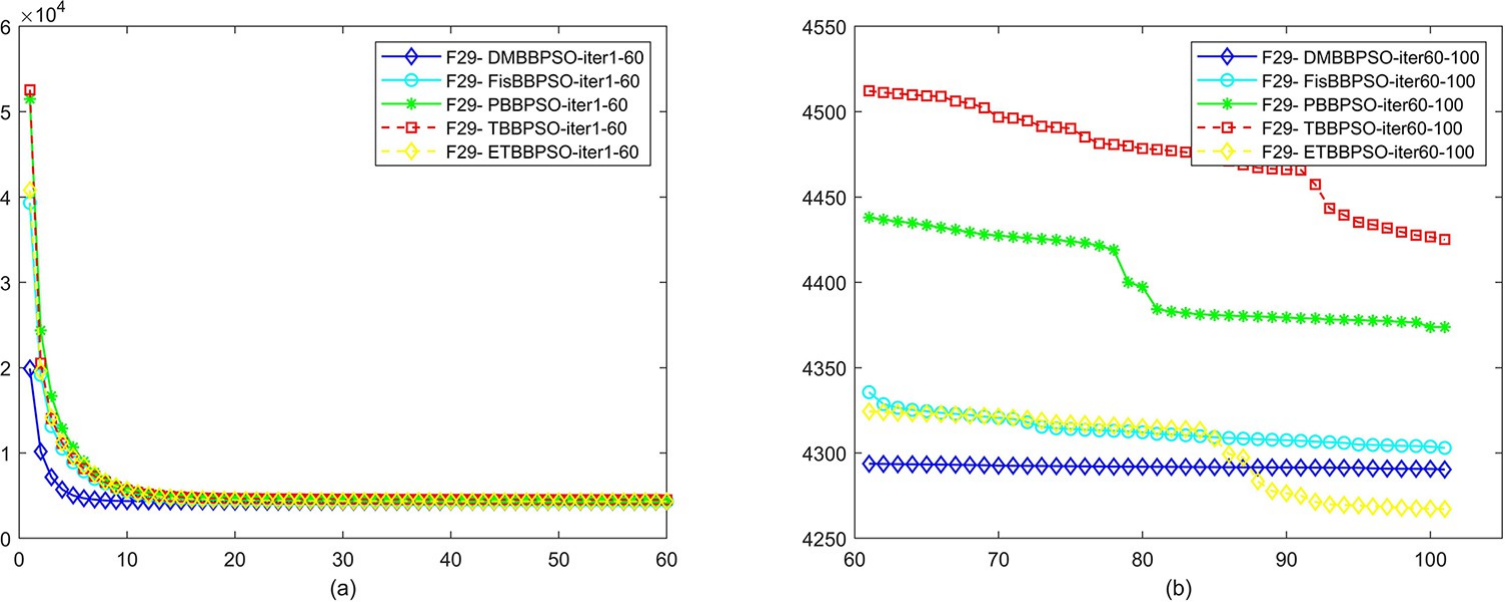
Comparison of convergence speed between DMBBPSO, FisBBPSO, PBBPSO, TBBPSO and ETBBPSO, *f*_29_. (a) iteration 0–6000, (b) iteration 6000–10000 the unit is 100 iterations.

To conclude, in a total of 29 benchmark functions, the DMBBPSO ranked first in 21 functions and ranked second in 3 functions. In addition, a rank competition is designed based on EEs. In each benchmark func-tion, the first, second, third, fourth and fifth functions will receive 1, 2, 3, 4 and 5 points. The average rank of DMBBPSO is 1.586, which own the best performance in the five algorithms.

The excellent optimization capability of the DMBBPSO is derived from the collaboration between MMSM and LAS. The MMSM catalyzes a set of deep memories to increase the diversity of the particle swarm. The LAS enables the particle swarm to avoid premature convergence while enhancing local search capabilities. Compared to traditional optimization tools, DMBBPSO does not require pre-training and parameter tuning. The simple structure and linear time complexity also allow DMBBPSO to be rapidly applied to a variety of practical applications.

Nevertheless, we find that DMBBPSO does not escape from the local optimum in all cases. We believe this is due to the fact that the current particle population does not possess enough memory depth to go back far enough in the past during the evolutionary process. On the other hand, blindly increasing the memory depth increases the computational effort, which leads to the algorithm running slowly.

Therefore, how to improve the performance of the algorithm while maintaining the computational speed is the main direction of future work. In addition, applying the evolutionary strategy of DMBBPSO to a multi-objective optimization algorithm is a feasible future work.

## Conclusions

A deep memory bare-bones particle swarm optimization algorithm (DMBBPSO) is proposed in this paper for single-objective optimization problems. The DMBBPSO improves the accuracy and stability of traditional BBPSO while maintaining linear time complexity. Compared to traditional optimization tools, DMBBPSO does not require pre-training and parameter tuning. The simple structure and linear time complexity also allow DMBBPSO to be rapidly applied to a variety of practical applications. Specifically, the DMBBPSO combines a multiple memory storage mechanisms (MMSM) and a layer-by-layer activation strategy (LAS). The MMSM enables an extra memory space for all particles, which is used to increase the diversity of every single particle. The cooperation of MMSM and LAS ensures the algorithm is able to implement high-precision local search while keeping a wide-range global search. Finally, simulation tests are implemented with the CEC 2017 benchmark functions. In a total of 29 benchmark functions, the DMBBPSO ranked first in 21 functions and ranked second in 3 functions. The average rank of DMBBPSO is 1.586, which own the best performance in the five algorithms. Experimental results confirmed that the DMBBPSO is able to present high precision results for single-objective optimization problems.
